# Supplementary material for: Impact evaluation of a cash-plus programme for children with disabilities in the Xiengkhouang Province in Lao PDR: study protocol for a non-randomised controlled trial
Source: BMJ Open. 2024 May 13;14(5):e081536. doi: 10.1136/bmjopen-2023-081536 (PMC11097848; doi:10.1136/bmjopen-2023-081536)
Supplement: Supplementary data [file bmjopen-2023-081536supp001.pdf]

**Domain**	**Measure of deprivation**
Education and early child development	Child (0-2): Child does not have any books/toys OR has inadequate supervision[1]<br>Child (3-5): Child is not in pre-primary/early childhood education OR has inadequate supervision<br>Child (6-17): Child is not attending school OR has missed more than 5 days of school in the last month
Health	All ages: Child has an unmet need for a health/rehabilitation service
Food security	Child (0-4): Child lives in a household that is food insecure<br>Child (5-17): Child lives in a household that is food insecure OR child reports being hungry but not eating because of lack of food in the last 30 days
Social participation	Child (0-4): Child has not been brought to a community/social event in which other children are present in the last month<br>Child (5-17): Child has at least one friend outside of their family OR did not play with friend(s) in the last 2 weeks
WASH[2]	Child (0-4): Child lives in a household without clean water source<br>Child (5-17): Child lives in a household without clean water sources OR child uses a different source than the rest of the family and this source is not a clean source OR child cannot access enough water to meet daily needs
	Child (0-4): Child lives in a household without an improved sanitation source<br>Child (5-17): Child lives in a household without an improved sanitation source sources OR child uses a different source than the rest of the family and this source is not improved OR child cannot use facility hygienically (i.e., without coming into contact with faeces, urine)
Violence	Child experiences any form of violence or discrimination in the past month

[1] Inadequate supervision defined as being left alone or under supervision of a child younger than 10 for more than one hour at least once in the last week (from UNICEF Multiple Cluster Indicator Surveys)

[2] Definitions of clean water and sanitation source according to SDG indicators (United Nations Statistic Division, 2021) and the Quality of WASH access questionnaire (Banks et al, 2019).
