# Supplementary material for: Impact evaluation of a cash-plus programme for children with disabilities in the Xiengkhouang Province in Lao PDR: study protocol for a non-randomised controlled trial
Source: BMJ Open. 2024 May 13;14(5):e081536. doi: 10.1136/bmjopen-2023-081536 (PMC11097848; doi:10.1136/bmjopen-2023-081536)
Supplement: Author's manuscript [file bmjopen-2023-081536.draft_revisions.pdf]

BMJ Open is committed to open peer review. As part of this commitment we make the peer review history of every article we publish publicly available.

When an article is published we post the peer reviewers' comments and the authors' responses online. We also post the versions of the paper that were used during peer review. These are the versions that the peer review comments apply to.

The versions of the paper that follow are the versions that were submitted during the peer review process. They are not the versions of record or the final published versions. They should not be cited or distributed as the published version of this manuscript.

BMJ Open is an open access journal and the full, final, typeset and author-corrected version of record of the manuscript is available on our site with no access controls, subscription charges or pay-per-view fees (http://bmjopen.bmj.com).

If you have any questions on BMJ Open's open peer review process please email info.bmjopen@bmj.com

BMJ Open

Impact evaluation of a cash-plus programme for children with disabilities in the Xiengkhouang Province in Lao PDR: Study protocol for a non-randomised control trial

Journal:	*BMJ Open*
Manuscript ID	bmjopen-2023-081536
Article Type:	Protocol
Date Submitted by the Author:	31-Oct-2023

Complete List of Authors:	Banks, Lena Morgon; London School of Hygiene & Tropical Medicine<br>Soukkhaphone, Bounhome; Laos Tropical & Public Health Institute<br>Scherer, Nathaniel; London School of Hygiene & Tropical Medicine<br>Siengsounthone, Latsamy; Laos Tropical & Public Health Institute<br>Carew, Mark T.; London School of Hygiene & Tropical Medicine<br>Shakespeare, Tom; London School of Hygiene and Tropical Medicine Faculty of Infectious and Tropical Diseases<br>Chen, Shanquan; London School of Hygiene & Tropical Medicine<br>Davey, Calum; London School of Hygiene and Tropical Medicine<br>Goyal, Divya; Independent consultant<br>Zinke, Anja; London School of Hygiene and Tropical Medicine, Department of Global Health & Development<br>Kuper, Hannah; The London School of Hygiene & Tropical Medicine, Clinical Research<br>Chanthakoumane, Ketmany; Laos Tropical & Public Health Institute

Keywords:	PUBLIC HEALTH, Community child health < PAEDIATRICS, Child protection < PAEDIATRICS

SCHOLARONE™
Manuscripts

Impact evaluation of a cash-plus programme for children with disabilities in the Xiengkhouang Province in Lao PDR: Study protocol for a non-randomised control trial

Lena Morgon Banks[1*Ω], Bounhome Soukkhaphone[2*], Nathaniel Scherer[1], Latsamy Siengsounthone[2], Mark T. Carew[1], Tom Shakespeare[1], Shanquan Chen[1], Calum Davey[1], Divya Goyal[3], Anja Zinke-Allmang[4], Hannah Kuper*[1], Ketmany Chanthakoumane[2*]

*These authors contributed equally to the work

[Ω]corresponding author: morgon.banks@lshtm.ac.uk

[1]International Centre for Evidence in Disability, London School of Hygiene & Tropical Medicine, London, United Kingdom, WC1E 7HT
[2]Laos Tropical & Public Health Institute, Vientiane, Laos
[3] Independent consultant, India
[4]Department of Population Health, London School of Hygiene & Tropical Medicine, London, United Kingdom, WC1E 7HT

For peer review only

Abstract

Introduction

More than 170 countries have implemented disability-targeted social protection programmes, although few have been rigorously evaluated. Consequently, a non-randomised control trial is being conducted of a pilot 'cash-plus' programme implemented by UNICEF Laos and the Laos government for children with disabilities in the the Xiengkhouang Province in Laos. The intervention combines a regular cash transfer with provision of assistive devices and access for caregivers to a family support programme.

Methods and analysis

The non-randomised control trial will involve 350 children with disabilities across 3 identified by programme implementers as eligible for the programme (intervention arm). Implementers have also identified approximately 180 children with disabilities in neighboring districts, who would otherwise meet eligibility criteria but do not live in the project areas (control arm) The trial will assess the impact of the programme on child well-being (primary outcome), as well as household poverty, caregiver quality of life and time use (secondary outcomes). Baseline data is being collected May-October 2023, with endline 24 months later. Analysis will be intention to treat. A complementary process evaluation will explore the acceptability of the programme, challenges and enablers to its delivery and mechanisms of impact.

Ethics and dissemination

The study has received ethical approval from the London School of Hygiene & Tropical Medicine and the National Ethics Committee for Health Research in Laos. Informed consent and assent will be taken by trained data collectors. Data will be collected and stored on a secure, encrypted server and its use will follow a detailed

For peer review only

data management plan. Findings will be disseminated in academic journals and in short briefs for policy and programmatic actors, and in online and in-person events.

Registration: This trial is registered with the ISRCTN registry (ISRCTN80603476) https://www.isrctn.com/ISRCTN80603476. It is protocol version 5 (June 20, 2023).

Keywords: disability, cash transfer, social protection, Laos, children with disabilities

For peer review only

Introduction

Social protection is an important tool for reducing poverty and protecting people from risks that affect well-being across the lifecycle (Kabeer and Waddington, 2015, McGuire et al., 2022). People with disabilities, who comprise 16% of the global population, are a core target group for social protection (World Health Organization, 2022, International Labour Organization, 2017). People with disabilities have a right to social protection under the United Nations Convention on the Rights of Persons with Disabilities (UNCRPD) which defines disability as including "… those who have long-term physical, mental, intellectual or sensory impairments which in interaction with various barriers may hinder their full and effective participation in society on an equal basis with others" (United Nations, 2006).

Further, people with disabilities may have a higher need for social protection. For example, they are more likely to be living in poverty compared to people without disabilities (Banks et al., 2017a, Pinilla-Roncancio and Alkire, 2021). They also frequently face "extra costs" for disability-related goods and services (e.g., rehabilitation, assistive devices, personal assistance) (Mont et al., 2022). As such, people with disabilities and their households often require a higher level of income than people without disabilities to meet both basic needs and disability-related costs (Mitra et al., 2017).

Many countries have recognised the need for and right to social protection amongst people with disabilities, with over 170 countries implementing disability-targeted social assistance programmes (International Labour Organization, 2017). However, there is a lack of evidence on the impact of social protection amongst people with

For peer review only

disabilities (Banks et al., 2016, Kidd et al., 2019). A systematic review on social protection and disability in low- and middle-income countries published in 2017 retrieved few rigorous studies on the impact of cash transfers and other programmes amongst people with disabilities, with available evidence limited to cross-sectional or qualitative studies of self-reported impacts with no impact evaluations (Banks et al., 2016). Since then, impact evaluations of cash transfers amongst people with disabilities have been conducted for programmes in the Maldives (disability-targeted), Malawi and Lesotho (both mainstream) (de Groot et al., 2021). However, the sample sizes of these studies were too small to disaggregate the impact of these programmes for children with disabilities for most outcome measures. Amongst people with disabilities of all ages, impacts of cash transfers were modest and mostly concentrated on improvements in food security, health status and increased health seeking behaviour (de Groot et al., 2021). Further evidence is needed on the impact of different programmes from different settings, and amongst children with disabilities in particular.

Additionally, there is an increasing recognition that cash transfers are an important but insufficient tool to push people out of poverty and improve their wellbeing (UNICEF, 2021). Cash transfers alone may be particularly insufficient for people with disabilities due to the poor availability, quality, accessibility and affordability of needed goods and services (Banks et al., 2021). As such, there is an increasing focus on developing integrated social protection or “cash plus” programmes. Cash plus programmes provide recipients with both a cash transfer as well as complementary programmes, such as health insurance and linkages to key services (Roelen et al., 2017). These programmes show particular promise for children and

For peer review only

adults with disabilities, who often face multiple barriers in improving their livelihoods and wellbeing (United Nations, 2019). People with disabilities report spending cash transfers primarily on health care (Banks et al., 2018a, Banks et al., 2018b), so cash plus programmes with entitlements linked to health are likely to be relevant.

Consequently, this study will conduct an impact evaluation of a cash plus programme targeted to children with disabilities in Laos. The programme combines a regular cash transfer with an assessment for and provision of rehabilitation and assistive devices. Caregivers are also invited to join a family support programme, to learn more about their child's disability and how to best support their child. The cash plus programme will be run by UNICEF Laos in partnership with the Laos government in three districts of the Xiengkhouang Province in Lao PDR. An accompanying process evaluation will also explore the acceptability of the programme, challenges and enablers to its delivery and mechanisms of impact.

Objectives

The overall aim of this research is to assess the impact of a cash-plus programme amongst children with disabilities and their households in Laos.

Specific objectives include:

- To evaluate the impact of a cash-plus programme on children's well-being
- To assess the impact of a cash-plus programme on caregivers' well-being
- To measure the impact of a cash-plus programme on household consumption expenditures
- To estimate the impact of a cash-plus programme on unmet needs for disability-related goods and services

For peer review only

- To explore the perceived impact of and satisfaction with the cash-plus programme amongst children with disabilities and their caregivers
- To examine challenges and enablers to the implementation and delivery of the cash-plus programme from the perspective of children/caregivers and implementers

Methods

Study design and settings

The trial will be a superiority, non-randomised control trial with two parallel groups. Three districts (Pek, Khoun, Phoukoud) in the Xiengkhouang Province were selected by UNICEF Laos and the Laos government to implement the cash-plus programme. Two other neighbouring districts were selected as control areas (Nonghed, Kham) after consultation between the research team (London School of Hygiene & Tropical Medicine – LSHTM, Laos Tropical & Public Health Institute – Laos TPHI) with UNICEF Laos as these areas have similar sociodemographic characteristics to the intervention districts.

A non-randomised control trial design was used as it was not possible to randomise children to receiving or not receiving the cash-plus programme, as the programme implementers had logistical constraints that meant they could only operate the programme in the selected areas at this time.

Baseline data is being conducted between May to October 2023. Collection of data

For peer review only

from the intervention group was completed before they began receiving any part of the programme. Allocation into the programme was known by implementers and the research team *a priori* although participants were not aware that they would be offered enrolment into the programme before baseline data collection. Delivery of the intervention began in August 2023, and parts (i.e. cash transfer) will be continued indefinitely. Endline data collection will take place 24 months from baseline.

Intervention description

The intervention includes a cash transfer along with an assessment for rehabilitation/assistive technology needs. Children found to have an unmet need for assistive technology will be provided with devices free of charge as part of the programme. Caregivers will also be invited to join a family support programme, which aims to improve their understanding of their child's disability and on how to provide support to their child.

The programme is being delivered by UNICEF Laos and the Laos government. They currently have funding to pilot the programme for approximately 450 children with disabilities in the Pek, Phoukoud and Khoun districts of Xiengkhouang Province. The intervention will continue to be offered to participants for the duration of the study period, unless the implementers face budgetary, political or other constraints in continuing to operate all or part of the programme. Participants are free to disenroll from the programme at any time. Participants in either arm are free to take up any other programme that is offered to them during the study period.

Eligibility criteria and recruitment

For peer review only

Data was collected from children with disabilities and their caregivers. To be eligible for this study, children had to be living in one of the study districts at the time of baseline data collection, be 18 years of age or younger and be identified by UNICEF Laos/Laos government as having a disability.

Participants (primarily caregivers of children with disabilities) were invited to participate in this study by members of the research team through either in-person or phone communication. Participants were provided with, or read out, an information sheet detailing the study protocol and risks and benefits of participating. Participants were provided with compensation to offset their time and other expenses (e.g., phone data) of taking part in the research.

Outcomes

Primary outcome measures

The primary outcome measure is child wellbeing, which will be measured across multiple dimensions. Multidimensional Overlapping Deprivation Analysis (MODA) have often been used for this purpose (de Neubourg et al., 2012). These indicators are also in line with Sustainable Development Goal (SDG) Indicator 1.2.2, which focuses on reducing poverty “in all its forms” (United Nations Statistics Division, 2021).

MODAs are indexes that collate several variables on deprivation. They can be tailored for the context and population group of interest (de Neubourg et al., 2012). The index for this study was created to be relevant to children with disabilities in Laos across the years of childhood (0-17 years) and covers domains that could be feasibly affected by receipt of the cash-plus programme. (Appendix 1). It was defined

For peer review only

through consultations with UNICEF Laos, Laos government and representatives of Organisations of Persons with Disabilities in Laos. Each indicator will be given equal weight such that the total of the index falls between 0 to 1 (0 being child experiences no deprivation on any indicator, 1 being child experiences deprivation across all indicators).

Secondary outcome measures

We will explore household consumption expenditures across food and non-food items. We will compare differences in overall consumption expenditures and by category (food, non-food, health, education). If possible, poverty will be measured by comparing consumption expenditures against the national poverty line. This measure is in line with SDG Indicator 1.2.1 (United Nations Statistics Division, 2021).

Caregiver well-being will be measured using the Pediatric Quality of Life (PedsQL) Family Impact Module, which looks at caregiver and family strain linked to having a child with a health condition (Varni et al., 2004). It has been used in different settings for measuring well-being of caregivers of children with disabilities (Duttine et al., 2022, Smythe et al., 2021). A score will be computed according to the tool's scoring guidance (Varni, 2014). Caregiver time use across different activities will be assessed using a modified version of the 'stylised activity list' developed for the Living Standard Measurement Survey (Harvey and Taylor, 2000).

Finally, we will assess unmet need for different disability-related goods and services by creating a module on the caregiver-reported need for and use of different goods and services required to support their child's participation (e.g. healthcare, assistive

For peer review only

devices, inclusive education supports, personal assistance). For each item, caregivers will be asked if they think their child needs the good/service, if they have gone for the service and their satisfaction with that service.

Sample size

The primary outcome measure is child well-being, measured through a MODA index. Using data from a previous study in Vietnam as parameters [15], we assume an average score of 0.4 (score out of 1) and a standard deviation of 0.12. With a sample size of 330 in the intervention arm and 180 in control, we can detect a minimum change in score of 0.035 (1/3 of a standard deviation) with power of 80%, type 1 error of 5% and 20% loss to follow-up.

Data collection and management

Baseline data will be collected between May-October 2023. Endline data will be collected 24 months later. All outcomes will be collected through a mobile survey survey created through Open Data Kit (ODK) to promote data quality. The survey tool has built-in skips and generates error messages for missing data and for non-logical answers (e.g., answers to daily time use questions exceeding 24 hours). The survey was developed by researchers at LSTHM and Laos TPHI, using validated tools (e.g., PedsQL, time use adapted from Living Standards Measurements Surveys) where possible. It was translated from English into Lao and Hmong. The translated versions were reviewed by multiple native speakers and pilot tested prior to implementation.

For peer review only

The survey will be administered by trained data collectors, who underwent a weeklong training in the study protocols, practice using the questionnaire and on how to promote quality data collection.

The child with disabilities' primary caregiver will be the main respondent, although children with disabilities ages 5-17 may answer certain modules (e.g., child's social participation, experiences in school). Other household members besides the primary caregiver may also contribute to sections on household consumption expenditures if they are the most knowledgeable household member. Multiple means of contacting participants were collected during baseline to decrease loss to follow-up by endline.

Data management

Data for the trial will be collected and stored on a secure and encrypted ODK server, hosted by LSHTM. The data will be cleaned and anonymised by an experienced data manager at Lao TPHI. Hard copies of data (e.g. written consent forms) will be kept in locked, secure cabinets at Laos TPHI. Electronic data (e.g., survey responses, interview recordings) will be kept on a password protected computer and on secure servers. A master file linking participants' names with ID numbers, signed consent forms, and contact/locator information will be stored in as an encrypted file on a password protected computer that is separate from the data files. Non-anonymised data will be destroyed 2 years after study completion.

Twenty-four months after the end of the project the data may be made available on LSHTM's Data Compass if it can be done so in a way that both allows for replication and does not lead to potential identification of participants. The possible identification

For peer review only

of participants is a concern given that a relatively small number of children are receiving the cash-plus programme: combining basic details such as age, gender, disability type and area of residence may be sufficient to identify a participant. The Data Protection Office at LSHTM will be consulted about the risk of identification.

If access to the data is possible, it will be made available upon request, provided the requester has a legitimate purpose for using the data. Requests will be managed by LSHTM's Data Compass team, which is independent to the research team.

Analysis

A detailed analysis plan will be published before the endline survey. The analysis will estimate intention-to-treat (ITT) effects.

We will assess the quality of the balance between control and intervention groups by describing the primary and secondary outcomes and sociodemographic variables at baseline. If there is evidence of imbalance, based on subjective interpretation of the magnitude of the difference, then we will plan *a priori* to include such variables in the main analysis of the primary and secondary outcomes.

We will estimate the effect of the intervention by comparing the proportions (e.g., proportion of children deprived on each domain of the MODA) and the means/medians (e.g., total MODA score, household consumption expenditures) between the arms of the trial. We will report the unadjusted estimated effects as risk ratios for binary outcomes and difference in the means for continuous outcomes. In the final analysis,

For peer review only

to increase the precision of the estimates and reduce the risk of bias from imbalances at baseline, we will use regression to adjust for the baseline levels of the outcome, stratification variables, and variables considered to be imbalanced at baseline. For binary outcomes we will model the risk ratio with a modified Poisson regression model (Zou, 2004). For continuous outcomes we will use linear regression. We will disaggregate findings by gender and other characteristics if the final sample sizes are sufficiently powered.

Methods in analysis to handle protocol non-adherence and any statistical methods to handle missing data

Analysis will be intention-to-treat. The programme implementers do not have plans to expand the delivery of the cash-plus programme to the control areas or to offer the programme to people newly moving into the intervention areas during the study period, so spill-over is unlikely. In the unlikely event that the programme is expanded to control areas, we may reduce the period of follow-up.

Missing data is expected to be minimal given the quality checks in the questionnaire (i.e., built-in skip patterns, error messages for unentered data). Some remaining missing data can be imputed. For example, for consumption expenditures data, missing items or items where participants could not give an accurate estimate of value will be imputed based on the median reported cost of that item from other households in the area.

Ethics and dissemination

For peer review only

Ethical approval has been received from the institutional review boards at the London School of Hygiene and Tropical Medicine in the United Kingdom (Reference: 28234) and from the National Ethics Committee for Health Research in Laos (Submission ID: 2023.03). Informed consent and assent will be taken by data collectors who received a weeklong training in the study protocols.

Informed consent and assent will be sought before the start of all interviews (i.e. survey, qualitative in-depth interviews). Consent will be sought from all adults (aged 18+) providing data. Caregivers who are married and between 14-17 years will also provide their own consent per Laos law and Laos ethics protocols. Data collection with children (aged 0-17, or 14-17 and unmarried) will require both child assent and caregiver consent.

For in-person data collection, the information sheet will be either read aloud or given to the participant to read and written consent will be sought (signature or thumbprint if illiterate). For remote data collection (e.g., by phone/video conferencing), an information sheet will be read aloud to participants. Oral consent will then be sought, and recorded (on a separate file to any interviews). Children will be read a simplified information sheet and will provide written or oral recorded assent (for in-person and remote interviews respectively). The informed consent and assent processes will make it clear that the decision to participate, to withdraw or to refuse to answer any questions will not have any negative consequences, including on any pre-existing or future services they are receiving from UNICEF, the Laos government or any other provider.

Collected data will be anonymised using a unique participant ID. A link log which links the unique ID to name will be kept on a secure server in a password protected file, separate from the data. All study staff working with the data have undergone

For peer review only

ethics training and will sign a confidentiality agreement. The study will comply with the General Data Protection Regulation, which requires that personal data must not be kept as identifiable data for longer than necessary for the purposes concerned. We will destroy all personally identifiable data two years after the end of the study.

Preliminary findings will be shared with UNICEF Laos, the Laos government and study participants for feedback. Findings from the trial will be published in academic journals and in short briefs targeted to a non-academic audience. Online and in-person dissemination events (e.g., conferences, webinars) will be conducted in Laos, the United Kingdom and elsewhere.

Discussion

The UNICEF Laos/Laos government cash-plus programme for children with disabilities is an important programme to evaluate. Importantly, little information exists on the impact of social protection, particularly cash-plus programmes, amongst people with disabilities (Banks et al., 2017b). Information is particularly lacking about such programmes amongst children, as studies are often underpowered to disaggregate by age (de Groot et al., 2021). However, access of children with disabilities and their families to social protection is particularly critical, as it may promote greater access to health, education and other support services that can have knock-on effects throughout their life-course. Information about the impact of this new, pilot programme, combined with a process evaluation detailing areas for strengthening, can help to inform improved delivery of this and other programmes in the future.

For peer review only

Still, there are some limitations of this evaluation. First, it was not feasible to conduct a non-randomised control trial. We will check for balance between the intervention and control arms and adjust analyses if there are differences between the two groups. However, it is possible that there are unobservable differences between arms that have not been accounted for. Second, some impacts of the programme may not be apparent after a 2-year follow-up period, particularly if there are delays in the roll-out of some components to the intervention. However, other evaluations of social protection programmes have found impacts on similar outcomes during this time frame (de Groot et al., 2021, Abdoulayi et al., 2016). Finally, some analyses, such as disaggregation by gender, may be underpowered depending on the final sample size.

Despite these possible limitations, this non-randomised control trial of a cash-plus programme will contribute to a limited evidence-base. Governments, international organisations and other actors have cited limited resources and evidence, as a barriers to investment in disability-targeted programmes. The lack of evidence on the effectiveness of social protection amongst people – particularly children – with disabilities hinders informed policymaking and planning, a gap which can begin to be filled this evaluation of a promising cash-plus programme.

For peer review only

Declarations

Acknowledgements

We are thankful to staff at UNICEF Laos, particularly Maryam Abdu and Amphayvan Chanmany for their contributions in informing the study design and identifying participants.

Author contributions

All authors contributed to the design of this protocol. LMB wrote the original draft of this paper and all other authors critically reviewed. LMB, BS, KC, NS, MTC, LS contributed to the development and review of study tools. HK obtained funding.

Funding

This research was funded by the United Kingdom's Foreign, Commonwealth and Development Office (PENDA project, grant PO8073). HK's salary is funded by an NIHR Global Research Professorship. LMB's time is partially covered by an Arts & Humanities networking grant (AH/X009580/1).

Availability of data and materials

We are investigating if it will be possible to anonymise the data in a way that would allow for reproduction of findings without identifying participants.

Ethics approval and consent to participate

Ethical approval has been received from the institutional review boards at the London School of Hygiene and Tropical Medicine in the United Kingdom (Reference: 28234) and from the National Ethics Committee for Health Research in Laos (Submission ID: 2023.03). Written or oral recorded informed consent will be obtained for all participants.

Competing interests

LMB has previously worked as a consultant for UNICEF and holds grants in which UNICEF is a collaborator. None of this work is linked to this evaluation and is through UNICEF headquarters rather than UNICEF Laos.

For peer review only

References

ABDOULAYI, S., GUSTAVO, A., BARRINGTON, C., BRUGH, K., HANDA, S., KILBURN, K., MOLOTSKY, A., OTCHERE, F., ZIETZ, S., MVULA, P., TSOKA, M., DE HOOP, J., PALERMO, T. & PETERMAN, A. 2016. Malawi Social Cash Transfer Programme Endline Impact Evaluation Report. Chapel Hill, North Carolina: Univeristy of North Carolina at Chapel Hill & University of Malawi.

BANKS, L. M., DAVEY, C., SHAKESPEARE, T. & KUPER, H. 2021. Disability-inclusive responses to COVID-19: Lessons learnt from research on social protection in low-and middle-income countries. *World development,* 137**,** 105178.

BANKS, L. M., KUPER, H. & POLACK, S. 2017a. Poverty and disability in low-and middle-income countries: A systematic review. *PloS one,* 12**,** e0189996.

BANKS, L. M., MEARKLE, R., MACTAGGART, I., WALSHAM, M., KUPER, H. & BLANCHET, K. 2016. Disability and social protection programmes in low-and middle-income countries: a systematic review. *Oxford Development Studies***,** 1-17.

BANKS, L. M., MEARKLE, R., MACTAGGART, I., WALSHAM, M., KUPER, H. & BLANCHET, K. 2017b. Disability and social protection programmes in low-and middle-income countries: a systematic review. *Oxford Development Studies,* 45**,** 223-239.

BANKS, L. M., WALSHAM, M., NEUPANE, S., NEUPANE, S., PRADHANANGA, Y., MAHARJAN, M., BLANCHET, K. & KUPER, H. 2018a. Disability-inclusive social protection research in Nepal: A national overview with a case study from Tanahun district. London: London School of Hygiene & Tropical Medicine.

BANKS, L. M., WALSHAM, M., VAN MINH, H., KIEN, V. D., MAI, V. Q., NGAN, T. T., PHUONG, B. B., SON, D. H., NGOC, N. B. & DUONG, D. T. T. 2018b. Disability-inclusive social protection research in Vietnam: A national overview with a case study from Cam Le district. London: London School of Hygiene & Tropical Medicine.

DE GROOT, R., PALERMO, T., BANKS, L. M. & KUPER, H. 2021. The impact of the Lesotho Child Grant Programme in the lives of children and adults with disabilities: Disaggregated analysis of a community randomized controlled trial. *International Social Security Review,* 74**,** 55-81.

DE NEUBOURG, C., CHAI, J., DE MILLIANO, M. & WEI, I. P. Z. 2012. Multiple Overlapping Deprivation Analysis (MODA).

DUTTINE, A., SMYTHE, T., DE SÁ, M. R. C., FERRITE, S., MOREIRA, M. E. & KUPER, H. 2022. Assessment of the feasibility of Juntos: A support programme for families of children affected by Congenital Zika Syndrome. *Wellcome Open Research,* 7.

HARVEY, A. S. & TAYLOR, M. E. 2000. Activity settings and travel behaviour: A social contact perspective. *Transportation,* 27**,** 53-73.

INTERNATIONAL LABOUR ORGANIZATION 2017. World Social Protection Report 2017-19: Universal social protection to achieve the Sustainable Development Goals. Geneva: ILO.

KABEER, N. & WADDINGTON, H. 2015. Economic impacts of conditional cash transfer programmes: a systematic review and meta-analysis. *Journal of Development Effectiveness,* 7**,** 290-303.

KIDD, S., WAPLING, L., SCHJOEDT, R., GELDERS, B., BAILEY-ATHIAS, D., TRAN, A. & SALOMON, H. 2019. Leaving no-one behind: Building inclusive social protection systems for persons with disabilities. Orpington, UK: Development Pathways.

MCGUIRE, J., KAISER, C. & BACH-MORTENSEN, A. M. 2022. A systematic review and meta-analysis of the impact of cash transfers on subjective well-being and mental health in low-and middle-income countries. *Nature Human Behaviour,* 6**,** 359-370.

MITRA, S., PALMER, M., KIM, H., MONT, D. & GROCE, N. 2017. Extra costs of living with a disability: A review and agenda for research. *Disability and health journal,* 10**,** 475-484.

MONT, D., COTE, A., HANASS-HANCOCK, J., BANKS, L. M., GRIGORUS, V., CARRARO, L., MORRIS, Z. & PINILLA-RONCANCIO, M. 2022. Estimating the Extra Costs for Disability for Social Protection Programs New York: UNPRPD.

PINILLA-RONCANCIO, M. & ALKIRE, S. 2021. How poor are people with disabilities? Evidence based on the global multidimensional poverty index. *Journal of Disability Policy Studies,* 31**,** 206-216.

ROELEN, K., DEVEREUX, S., ABDULAI, A.-G., MARTORANO, B., PALERMO, T. & RAGNO, L. P. 2017. How to make 'cash plus' work: Linking cash transfers to services and sectors.

SMYTHE, T., REICHENBERGER, V., PINZÓN, E. M., HURTADO, I. C., RUBIANO, L. & KUPER, H. 2021. The feasibility of establishing parent support groups for children with congenital Zika syndrome and their families: a mixed-methods study. *Wellcome Open Research,* 6**,** 158.

UNICEF. 2021. *Cash Plus* [Online]. Available: https://www.unicef-irc.org/research/cash-plus/ [Accessed September 10 2022].

UNITED NATIONS 2006. United Nations Convention on the Rights of Persons with Disabilities. *In:* UNITED NATIONS (ed.) *Article 1.*

UNITED NATIONS 2019. Disability and Development Report 2018. *In:* AFFAIRS, D. O. E. A. S. (ed.). New York.

UNITED NATIONS STATISTICS DIVISION. 2021. *SDG indicators: Metadata repository* [Online]. Available: https://unstats.un.org/sdgs/metadata/ [Accessed June 23 2021].

VARNI, J. W. 2014. Scaling and Scoring of the Pediatric Quality of Life Inventory™ (PedsQL). Lyon, France: Mapi Research Trust.

VARNI, J. W., SHERMAN, S. A., BURWINKLE, T. M., DICKINSON, P. E. & DIXON, P. 2004. The PedsQL™ family impact module: preliminary reliability and validity. *Health and quality of life outcomes,* 2**,** 1-6.

WORLD HEALTH ORGANIZATION 2022. *Global report on health equity for persons with disabilities,* Geneva, World Health Organization.

ZOU, G. 2004. A modified poisson regression approach to prospective studies with binary data. *American journal of epidemiology,* 159**,** 702-706.

For peer review only

Domain	Measure of deprivation
Education and early child development	Child (0-2): Child does not have any books/toys OR has inadequate supervision[1]<br>Child (3-5): Child is not in pre-primary/early childhood education OR has inadequate supervision<br>Child (6-17): Child is not attending school OR has missed more than 5 days of school in the last month
Health	All ages: Child has an unmet need for a health/rehabilitation service
Food security	Child (0-4): Child lives in a household that is food insecure<br>Child (5-17): Child lives in a household that is food insecure OR child reports being hungry but not eating because of lack of food in the last 30 days
Social participation	Child (0-4): Child has not been brought to a community/social event in which other children are present in the last month<br>Child (5-17): Child has at least one friend outside of their family OR did not play with friend(s) in the last 2 weeks
WASH[2]	Child (0-4): Child lives in a household without clean water source<br>Child (5-17): Child lives in a household without clean water sources OR child uses a different source than the rest of the family and this source is not a clean source OR child cannot access enough water to meet daily needs
	Child (0-4): Child lives in a household without an improved sanitation source<br>Child (5-17): Child lives in a household without an improved sanitation source sources OR child uses a different source than the rest of the family and this source is not improved OR child cannot use facility hygienically (i.e., without coming into contact with faeces, urine)
Violence	Child experiences any form of violence or discrimination in the past month

[1] Inadequate supervision defined as being left alone or under supervision of a child younger than 10 for more than one hour at least once in the last week (from UNICEF Multiple Cluster Indicator Surveys)

[2] Definitions of clean water and sanitation source according to SDG indicators (United Nations Statistic Division, 2021) and the Quality of WASH access questionnaire (Banks et al, 2019).

For peer review only

BMJ Open

Impact evaluation of a cash-plus programme for children with disabilities in the Xiengkhouang Province in Lao PDR: Study protocol for a non-randomised control trial

Journal:	*BMJ Open*
Manuscript ID	bmjopen-2023-081536.R1
Article Type:	Protocol
Date Submitted by the Author:	08-Mar-2024
Complete List of Authors:	Banks, Lena Morgon; London School of Hygiene & Tropical Medicine<br>Soukkhaphone, Bounhome; Laos Tropical & Public Health Institute<br>Scherer, Nathaniel; London School of Hygiene & Tropical Medicine<br>Siengsounthone, Latsamy; Laos Tropical & Public Health Institute<br>Carew, Mark T.; London School of Hygiene & Tropical Medicine<br>Shakespeare, Tom; London School of Hygiene and Tropical Medicine Faculty of Infectious and Tropical Diseases<br>Chen, Shanquan; London School of Hygiene & Tropical Medicine<br>Davey, Calum; London School of Hygiene and Tropical Medicine<br>Goyal, Divya; Independent consultant<br>Zinke, Anja; London School of Hygiene and Tropical Medicine, Department of Global Health & Development<br>Kuper, Hannah; The London School of Hygiene & Tropical Medicine, Clinical Research<br>Chanthakoumane, Ketmany; Laos Tropical & Public Health Institute
**Primary Subject Heading**:	Global health
Secondary Subject Heading:	Evidence based practice
Keywords:	PUBLIC HEALTH, Community child health < PAEDIATRICS, Disabled Persons, Social Support, Rehabilitation medicine < INTERNAL MEDICINE

SCHOLARONE™
Manuscripts

Abstract

Introduction

More than 170 countries have implemented disability-targeted social protection programmes, although few have been rigorously evaluated. Consequently, a non-randomised control trial is being conducted of a pilot 'cash-plus' programme implemented by UNICEF Laos and the Laos government for children with disabilities in the the Xiengkhouang Province in Laos. The intervention combines a regular cash transfer with provision of assistive devices and access for caregivers to a family support programme.

Methods and analysis

The non-randomised control trial will involve 350 children with disabilities across 3 districts identified by programme implementers as eligible for the programme (intervention arm). Implementers have also identified approximately 180 children with disabilities in neighboring districts, who would otherwise meet eligibility criteria but do not live in the project areas (control arm) The trial will assess the impact of the programme on child well-being (primary outcome), as well as household poverty, caregiver quality of life and time use (secondary outcomes). Baseline data is being collected May-October 2023, with endline 24 months later. Analysis will be intention to treat. A complementary process evaluation will explore the implementation, acceptability of the programme, challenges and enablers to its delivery and mechanisms of impact.

Ethics and dissemination

The study has received ethical approval from the London School of Hygiene & Tropical Medicine and the National Ethics Committee for Health Research in Laos. Informed consent and assent will be taken by trained data collectors. Data will be

For peer review only

collected and stored on a secure, encrypted server and its use will follow a detailed data management plan. Findings will be disseminated in academic journals and in short briefs for policy and programmatic actors, and in online and in-person events.

Strengths and limitations of this study:

- This study will be one of the few impact evaluations of a cash-plus programme targeted to children with disabilities, which can be used to inform decisions about not just the design and delivery of this programme in Laos but will have learnings for other programmes in other settings as well.
- The cash-plus programme under study is novel and addresses diverse drivers of poverty and decreased well-being amongst children with disabilities and their households.
- The study design is robust and combines a non-randomised control trial and a process evaluation.
- Still, individuals could not be randomised and so there may be unobservable differences between intervention and control arms.
- Similarly, the sample size likely will not have sufficient power to explore meaningful differences amongst children with disabilities (e.g., if impacts differ by the child's gender, disability type etc).

Registration: This trial is registered with the ISRCTN registry (ISRCTN80603476) https://www.isrctn.com/ISRCTN80603476. It is protocol version 5 (June 20, 2023).

Keywords: disability, cash transfer, social protection, Laos, children with disabilities

For peer review only

Introduction

Social protection is an important tool for reducing poverty and protecting people from risks that affect well-being across the lifecycle [1, 2]. People with disabilities, who comprise 16% of the global population, are a core target group for social protection [3, 4]. People with disabilities have a right to social protection under the United Nations Convention on the Rights of Persons with Disabilities (UNCRPD) which defines disability as including "… those who have long-term physical, mental, intellectual or sensory impairments which in interaction with various barriers may hinder their full and effective participation in society on an equal basis with others" [5].

Further, people with disabilities may have a higher need for social protection. For example, they are more likely to be living in poverty compared to people without disabilities [6, 7]. People with disabilities are often excluded from opportunities for decent work, due to discrimination or earlier barriers to receiving quality education and training, while caregivers often reduce time on income-generating activities for additional caretaking responsibilities [8]. They also frequently face "extra costs" for disability-related goods and services (e.g., rehabilitation, assistive devices, personal assistance) [9]. As such, people with disabilities and their households often require a higher level of income than people without disabilities to meet both basic needs and disability-related costs [10].

Many countries have recognised the need for and right to social protection amongst people with disabilities, with over 170 countries implementing disability-targeted social assistance programmes [4]. The coverage and content of these programmes

For peer review only

can vary widely: for example, only 9% of people with severe disabilities receive a disability benefit in low-income countries compared to 86% of people with severe disabilities in high income countries [11]. However, there is a lack of evidence on the impact of social protection amongst people with disabilities [12, 13]. A systematic review on social protection and disability in low- and middle-income countries published in 2017 retrieved few rigorous studies on the impact of cash transfers and other programmes amongst people with disabilities, with available evidence limited to cross-sectional or qualitative studies of self-reported impacts with no impact evaluations [12]. Since then, impact evaluations of cash transfers amongst people with disabilities have been conducted for disability-targeted programmes in the Maldives and China [14-16], as well as mainstream programmes in Malawi and Lesotho [17]. Amongst people with disabilities of all ages, impacts of cash transfers were modest and mostly concentrated on improvements in food security, health status and increased health seeking behaviour [14, 17]. Further evidence is needed on the impact of different programmes from different settings, and amongst children with disabilities and their households in particular.

Additionally, there is an increasing recognition that cash transfers are an important but insufficient tool to push people out of poverty and improve their wellbeing [18]. Cash transfers alone may be particularly insufficient for people with disabilities and their households due to the poor availability, quality, accessibility and affordability of needed goods and services [19]. For example, assistive devices – such as wheelchairs, prosthetics, hearing aids, walking canes – are essential for improving people with disabilities' functioning and participation; however, in many settings, people with disabilities lack access to these devices because their supply is very

For peer review only

limited, they lack information about their utility and where to access them and available devices are of poor quality [20]. Additionally, children with disabilities and their households may face other barriers to improving their wellbeing, such as discrimination and negative attitudes on disability as well as lack of inclusive health, education and other services [8]. Caregivers may lack knowledge about disability and how to best support their child with a disability, which can lead to increased caregiver stress, less effective caregiving and ultimately poorer child outcomes [21].

As such, there is an increasing focus on developing integrated social protection or "cash plus" programmes. Cash plus programmes provide recipients with both a cash transfer as well as complementary programmes, such as health insurance and linkages to key services [22]. These programmes show particular promise for children and adults with disabilities, who often face multiple barriers in improving their livelihoods and wellbeing [8]. People with disabilities report spending cash transfers primarily on health care [23, 24], so cash plus programmes with entitlements linked to health are likely to be relevant. In China, receipt of a disability-targeted cash transfer was linked to an increased likelihood of using rehabilitation and medical services amongst both children and adults with disabilities [15, 16].

Consequently, this study will conduct an impact evaluation of a cash plus programme targeted to children with disabilities in Laos. According to the 2015 Population and Housing Census, 2.8% of the population over 5 years of age in Laos is living with a disability, with prevalence higher in remote areas [25]. The Lao PDR Constitution and the Law on Persons with Disabilities stipulates equal protections of the rights of people with disabilities, and the latter calls for the creation of a welfare fund for people with disabilities [26]. Overall, Laos' social protection system is nascent, but growing. For example, health insurance coverage expanded rapidly

For peer review only

from 11% in 2008 to 93% currently [27]. It covers a range of preventative, promotive, curative and some rehabilitation services. However, it is acknowledged that insufficient state financing of the health system has led to inadequate services and high out-of-pocket payments [27]. Laos does not currently have comprehensive social protection programmes for people with disabilities, although expanding services and benefits to this group is listed as priority for action in the most recent National Social Protection Strategy [26].

This impact evaluation will assess a pilot of one such programme. The pilot programme combines a regular cash transfer with an assessment for and provision of rehabilitation and assistive devices. Caregivers are also invited to join a family support programme, which focuses on improving their livelihood skills and on learning more about disability and how to best support their child with a disability. The cash plus programme will be run by UNICEF Laos in partnership with the Laos government in three districts of the Xiengkhouang Province in Lao PDR. An accompanying process evaluation will also explore the implementation process, acceptability of the programme, challenges and enablers to its delivery and mechanisms of impact. This information will be critical for the Lao PDR government, given their prioritisation of disability within strategies for scaling-up the national social protection system [26]. It will also have implications for other contexts, given the lack of evaluations on disability-targeted cash-plus programmes.

Objectives

The overall aim of this research is to assess the impact of a cash-plus programme amongst children with disabilities and their households in Laos.

Specific objectives include:

- To evaluate the impact of a cash-plus programme on children's well-being
- To assess the impact of a cash-plus programme on caregivers' well-being
- To measure the impact of a cash-plus programme on household consumption expenditures
- To estimate the impact of a cash-plus programme on unmet needs for disability-related goods and services
- To explore the perceived impact, and acceptability of the cash-plus programme amongst children with disabilities and their caregivers
- To examine the implementation process, challenges and enablers to the delivery and mechanisms of impact of the cash-plus programme from the perspective of children/caregivers and implementers

Methods

Study design and settings

The trial will be a superiority, non-randomised control trial with two parallel groups. Three districts (Pek, Khoun, Phoukoud) in the Xiengkhouang Province were selected by UNICEF Laos and the Laos government to implement the cash-plus programme. Two other neighbouring districts were selected as control areas (Nonghed, Kham) after consultation between the research team (London School of Hygiene & Tropical Medicine – LSHTM, Laos Tropical & Public Health Institute – Laos TPHI) with UNICEF Laos as these areas have similar sociodemographic characteristics to the intervention districts.

A non-randomised control trial design was used as it was not possible to randomise

For peer review only

children to receiving or not receiving the cash-plus programme, as the programme implementers had logistical constraints that meant they could only operate the programme in the selected areas at this time.

Baseline data is being conducted between May to October 2023. Collection of data from the intervention group was completed before they began receiving any part of the programme. Allocation into the programme was known by implementers and the research team *a priori* although participants were not aware that they would be offered enrolment into the programme before baseline data collection. Delivery of the intervention began in September 2023, and parts (i.e. cash transfer) will be continued indefinitely funding dependent. Endline data collection will take place 24 months from baseline. The process evaluation will be conducted in 2024 after the delivery of the family support programme and assistive device components.

Intervention description

The intervention includes a cash transfer along with an assessment for assistive devices (e.g., wheelchair, prosthesis, hearing aid, walking cane). Children found to have an unmet need for assistive devices will be provided with devices free of charge as part of the programme. Caregivers will also be invited to join a family support programme, which aims to improve their understanding of their child's disability and on how to provide support to their child. Figure 1 presents a theory of change for how the intervention components are expected to improve child and caregiver well-being.

Figure 1. Theory of change for cash-plus programme

For peer review only

The programme is being delivered by UNICEF Laos and the Laos government. They currently have funding to pilot the programme for approximately 450 children with disabilities in the Pek, Phoukoud and Khoun districts of Xiengkhouang Province. The intervention is expected to continue to be offered to participants for the duration of the study period. This timeline may change if implementers face budgetary, political or other constraints in continuing to operate all or part of the programme. Funding is guaranteed until June 2024, and will be reassessed for continuation before then. Participants are free to disenroll from the programme at any time. Participants in either arm are free to take up any other programme that is offered to them during the study period.

Eligibility criteria and recruitment

Data was collected from children with disabilities and their caregivers. To be eligible for this study, children had to be living in one of the study districts at the time of baseline data collection, be 18 years of age or younger and be identified by UNICEF Laos/Laos government as having a disability. Disability determination and identification for both the intervention and control groups was conducted by UNICEF Laos. An initial sample – which strove to identify all children with disabilities in the study areas – involved a combination of past survey data shared by the government of Laos and identification conducted by community leaders; these children were then verified by UNICEF to ensure they had a disability and were of the target age. Further details on the identification process will be explored as part of the process evaluation.

For the process evaluation, approximately 15 caregivers of children with disabilities from the intervention group will be interviewed. They will be selected to maximise

For peer review only

heterogeneity by the child's gender, disability type, age and location. Additionally, about 12 programme implementers will be interviewed. They will be selected to reflect diversity of roles in the design and delivery of the programme.

Participants (primarily caregivers of children with disabilities) were invited to participate in this study by members of the research team through either in-person or phone communication. Participants were provided with, or read out, an information sheet detailing the study protocol and risks and benefits of participating. Participants were provided with compensation to offset their time and other expenses (e.g., phone data) of taking part in the research.

Outcomes

Primary outcome measures

The primary outcome measure is child wellbeing, which will be measured across multiple dimensions. Multidimensional Overlapping Deprivation Analysis (MODA) have often been used for this purpose [28]. These indicators are also in line with Sustainable Development Goal (SDG) Indicator 1.2.2, which focuses on reducing poverty "in all its forms" [29].

MODAs are indexes that collate several variables on deprivation. They can be tailored for the context and population group of interest [28]. The index for this study was created to be relevant to children with disabilities in Laos across the years of childhood (0-17 years) and covers domains that could be feasibly affected by receipt of the cash-plus programme. (Appendix 1). It included commonly used measures, such as ones used in the Multiple Indicator Cluster Surveys that have been administered in over 100 countries including Laos. It was defined through

For peer review only

consultations with UNICEF Laos, Laos government and representatives of Organisations of Persons with Disabilities in Laos. Each indicator will be given equal weight such that the total of the index falls between 0 to 1 (0 being child experiences no deprivation on any indicator, 1 being child experiences deprivation across all indicators).

Secondary outcome measures

We will explore household consumption expenditures across food and non-food items. We will compare differences in overall consumption expenditures and by category (food, non-food, health, education). If possible, poverty will be measured by comparing consumption expenditures against the national poverty line. This measure is in line with SDG Indicator 1.2.1 [29].

Caregiver well-being will be measured using the Pediatric Quality of Life (PedsQL) Family Impact Module, which looks at caregiver and family strain linked to having a child with a health condition [30]. It has been used in different settings for measuring well-being of caregivers of children with disabilities [31, 32]. A score will be computed according to the tool's scoring guidance [33]. Caregiver time use across different activities will be assessed using a modified version of the 'stylised activity list' developed for the Living Standard Measurement Survey [34].

Finally, we will assess unmet need for different disability-related goods and services by creating a module on the caregiver-reported need for and use of different goods and services required to support their child's participation (e.g. healthcare, assistive devices, inclusive education supports, personal assistance). For each item,

For peer review only

caregivers will be asked if they think their child needs the good/service, if they have gone for the service and their satisfaction with that service.

Process evaluation

The process evaluation will follow the Medical Research Council (MRC) framework [35]. It will examine the structures and resources used to deliver the intervention and what was delivered, for example in terms of whether the intervention reached all whom it was designed to reach.

It will also explore mechanisms of impact, including participants' reflections on the strengths and challenges of different components and their perceptions of the impact of the programme (or lack thereof) on their lives, children and personal wellbeing. The latter will complement the quantitative measures of impact, as concepts such as well-being are complex and challenging to measure. Finally, the process evaluation will explore the role of context, for example how underlying socioeconomic factors, social attitudes to disability and broader disability inclusion in the study areas may have influenced the effectiveness of the programme.

Sample size

The primary outcome measure is child well-being, measured through a MODA index. Disability-sensitive MODAs are lacking, but using data from a previous study in Vietnam as parameters [15], we assume an average score of 0.4 (score out of 1) and a standard deviation of 0.12. With a sample size of 330 in the intervention arm and 180 in control, we can detect a minimum change in score of 0.035 (1/3 of a standard deviation) with power of 80%, type 1 error of 5% and 20% loss to follow-up.

For peer review only

The process evaluation will include in-depth interviews with about 15 caregivers of children with disabilities and 12 implementers.

Data collection and management

Baseline data will be collected between May-October 2023. Endline data will be collected 24 months later. All outcomes will be collected through a mobile survey survey created through Open Data Kit (ODK) to promote data quality. The survey tool has built-in skips and generates error messages for missing data and for non-logical answers (e.g., answers to daily time use questions exceeding 24 hours). The survey was developed by researchers at LSTHM and Laos TPHI, using validated tools (e.g., PedsQL, time use adapted from Living Standards Measurements Surveys) where possible. It was translated from English into Lao and Hmong. The translated versions were reviewed by multiple native speakers and pilot tested prior to implementation.

The survey will be administered by trained data collectors, who underwent a weeklong training in the study protocols, practice using the questionnaire and on how to promote quality data collection. They are predominantly public health students at the Lao TPHI and speak Lao and/or Hmong.

The child with disabilities' primary caregiver will be the main respondent, although children with disabilities ages 5-17 may answer certain modules (e.g., child's social participation, experiences in school). Other household members besides the primary caregiver may also contribute to sections on household consumption expenditures if they are the most knowledgeable household member. Multiple means of contacting participants were collected during baseline to decrease loss to follow-up by endline.

For peer review only

The process evaluation will be conducted in 2024 after the completion of the family support programme and delivery of assistive devices. In-depth interviews with caregivers and implementers will be conducted by trained interviewers in Lao, Hmong or English depending on the preference of the participant. They will use semi-structured interview guides. For implementers, these guides will be adapted to reflect the expertise and role of the respondent.

Data management

Data for the trial will be collected and stored on a secure and encrypted ODK server, hosted by LSHTM. In-depth interviews will be recorded, translated and transcribed. All data will be cleaned and anonymised by an experienced data manager at Lao TPHI. Hard copies of data (e.g. written consent forms) will be kept in locked, secure cabinets at Laos TPHI. Electronic data (e.g., survey responses, interview recordings) will be kept on a password protected computer and on secure servers. A master file linking participants' names with ID numbers, signed consent forms, and contact/locator information will be stored in as an encrypted file on a password protected computer that is separate from the data files. Non-anonymised data will be destroyed 2 years after study completion.

Twenty-four months after the end of the project the data may be made available on LSHTM's Data Compass if it can be done so in a way that both allows for replication and does not lead to potential identification of participants. The possible identification of participants is a concern given that a relatively small number of children are receiving the cash-plus programme: combining basic details such as age, gender,

For peer review only

disability type and area of residence may be sufficient to identify a participant. The Data Protection Office at LSHTM will be consulted about the risk of identification.

If access to the data is possible, it will be made available upon request, provided the requester has a legitimate purpose for using the data. Requests will be managed by LSHTM's Data Compass team, which is independent to the research team.

Patient and public involvement

Preliminary outcome indicators were checked with the Lao Disabled People's Association, an Organisation of Persons with Disabilities, to confirm relevance for people with disabilities in Laos. Findings from the study will be disseminated back to participants (e.g., through community events).

Analysis

A detailed analysis plan will be published before the endline survey. The analysis will estimate intention-to-treat (ITT) effects.

We will assess the quality of the balance between control and intervention groups by describing the primary and secondary outcomes and sociodemographic variables at baseline. If there is evidence of imbalance, based on subjective interpretation of the magnitude of the difference, then we will plan *a priori* to include such variables in the main analysis of the primary and secondary outcomes.

We will estimate the effect of the intervention by comparing the proportions (e.g., proportion of children deprived on each domain of the MODA) and the means/medians

For peer review only

(e.g., total MODA score, household consumption expenditures) between the arms of the trial. We will report the unadjusted estimated effects as risk ratios for binary outcomes and difference in the means for continuous outcomes. In the final analysis, to increase the precision of the estimates and reduce the risk of bias from imbalances at baseline, we will use regression to adjust for the baseline levels of the outcome, stratification variables, and variables considered to be imbalanced at baseline. For binary outcomes we will model the risk ratio with a modified Poisson regression model [36]. For continuous outcomes we will use linear regression. We will disaggregate findings by gender and other characteristics if the final sample sizes are sufficiently powered.

For the process evaluation, thematic analysis will be used (Braun & Clarke, 2006). Transcripts of in-depth interviews will be analysed both deductively and inductively in NVivo. Deductive analysis will use a coding framework based on the core areas of the MRC process evaluation framework, with inductive coding being used to capture additional themes identified by analysts in the data. Analysis will consider differences by sub-groups, such as by the gender or disability type of the child.

Methods in analysis to handle protocol non-adherence and any statistical methods to handle missing data

Analysis will be intention-to-treat. The programme implementers do not have plans to expand the delivery of the cash-plus programme to the control areas or to offer the programme to people newly moving into the intervention areas during the study period, so spill-over is unlikely. In the unlikely event that the programme is expanded to control areas, we may reduce the period of follow-up.

For peer review only

Missing data is expected to be minimal given the quality checks in the questionnaire (i.e., built-in skip patterns, error messages for unentered data). Some remaining missing data can be imputed. For example, for consumption expenditures data, missing items or items where participants could not give an accurate estimate of value will be imputed based on the median reported cost of that item from other households in the area.

Ethics and dissemination

Ethical approval has been received from the institutional review boards at the London School of Hygiene and Tropical Medicine in the United Kingdom (Reference: 28234) and from the National Ethics Committee for Health Research in Laos (Submission ID: 2023.03). Any amendments will be approved by these bodies, and uploaded into the trial registry (https://www.isrctn.com/ISRCTN80603476).

Informed consent and assent will be sought before the start of all interviews (i.e. survey, qualitative in-depth interviews) by trained data collectors. Consent will be sought from all adults (aged 18+) providing data. Caregivers who are married and between 14-17 years will also provide their own consent per Laos law and Laos ethics protocols. Data collection with children (aged 0-17, or 14-17 and unmarried) will require both child assent and caregiver consent.

Ethical approval was granted for both in-person and remote data collection and consent taking. In-person data collection is the main and preferred strategy. However, remote data collection is needed in some instances as severe weather events and poor roads limited access by the research team to participants. So far, response rates for remote data collection have been adequate. For in-person data

For peer review only

collection, the information sheet will be either read aloud or given to the participant to read and written consent will be sought (signature or thumbprint if illiterate). For remote data collection (e.g., by phone/video conferencing), an information sheet will be read aloud to participants. Oral consent will then be sought, and recorded (on a separate file to any interviews). Children will be read a simplified information sheet and will provide written or oral recorded assent (for in-person and remote interviews respectively). The informed consent and assent processes will make it clear that the decision to participate, to withdraw or to refuse to answer any questions will not have any negative consequences, including on any pre-existing or future services they are receiving from UNICEF, the Laos government or any other provider. Further, participants' responses and their decision to participate or not will not be shared with UNICEF, the government or anyone outside of the research team.

Collected data will be anonymised using a unique participant ID. A link log which links the unique ID to name will be kept on a secure server in a password protected file, separate from the data. All study staff working with the data have undergone ethics training and will sign a confidentiality agreement. The study will comply with the General Data Protection Regulation, which requires that personal data must not be kept as identifiable data for longer than necessary for the purposes concerned. We will destroy all personally identifiable data two years after the end of the study.

Preliminary findings will be shared with UNICEF Laos, the Laos government and study participants for feedback. Findings from the trial will be published in academic journals and in short briefs targeted to a non-academic audience. Online and in-person dissemination events (e.g., conferences, webinars) will be conducted in Laos, the United Kingdom and elsewhere.

For peer review only

Discussion

The UNICEF Laos/Laos government cash-plus programme for children with disabilities is an important programme to evaluate. Importantly, little information exists on the impact of social protection, particularly cash-plus programmes, amongst people with disabilities [37]. Information is particularly lacking about such programmes amongst children, as studies are often underpowered to disaggregate by age [17]. However, access of children with disabilities and their families to social protection is particularly critical, as it may promote greater access to health, education and other support services that can have knock-on effects throughout their life-course. Information about the impact of this new, pilot programme, combined with a process evaluation detailing areas for strengthening, can help to inform improved delivery of this and other programmes in the future.

Still, there are some limitations of this evaluation. First, it was not feasible to conduct a non-randomised control trial. We will check for balance between the intervention and control arms and adjust analyses if there are differences between the two groups. However, it is possible that there are unobservable differences between arms that have not been accounted for. Second, some impacts of the programme may not be apparent after a 2-year follow-up period, particularly if there are delays in the roll-out of some components to the intervention. However, other evaluations of social protection programmes have found impacts on similar outcomes during this time frame [17, 38]. Finally, some analyses, such as disaggregation by gender, may be underpowered depending on the final sample size.

For peer review only

Despite these possible limitations, this non-randomised control trial of a cash-plus programme will contribute to a limited evidence-base. Governments, international organisations and other actors have cited limited resources and evidence, as a barriers to investment in disability-targeted programmes. The lack of evidence on the effectiveness of social protection amongst people – particularly children – with disabilities hinders informed policymaking and planning, a gap which can begin to be filled this evaluation of a promising cash-plus programme.

Declarations

Acknowledgements

We are thankful to staff at UNICEF Laos, particularly Maryam Abdu and Amphayvan Chanmany for their contributions in informing the study design and identifying participants.

Author contributions

LMB, BS, NS, LS, MTC, TS, SC, CD, DG, AZA, HK, KC contributed to the design of this protocol. LMB wrote the original draft of this paper and BS, NS, LS, MTC, TS, SC, CD, DG, AZA, HK, KC critically reviewed. LMB, BS, KC, NS, MTC, LS contributed to the development and review of study tools. HK obtained funding.

Funding

This research was funded by the United Kingdom's Foreign, Commonwealth and Development Office (PENDA project, grant PO8073). HK's salary is funded by an NIHR Global Research Professorship. LMB's time is partially covered by an Arts & Humanities networking grant (AH/X009580/1) and the Open Access fee is covered by this grant.

For peer review only

Availability of data and materials

We are investigating if it will be possible to anonymise the data in a way that would allow for reproduction of findings without identifying participants.

Ethics approval and consent to participate

Ethical approval has been received from the institutional review boards at the London School of Hygiene and Tropical Medicine in the United Kingdom (Reference: 28234) and from the National Ethics Committee for Health Research in Laos (Submission ID: 2023.03). Written or oral recorded informed consent will be obtained for all participants.

Competing interests

LMB has previously worked as a consultant for UNICEF and holds grants in which UNICEF is a collaborator. None of this work is linked to this evaluation and is through UNICEF headquarters rather than UNICEF Laos.

References

1. Kabeer, N. and H. Waddington, *Economic impacts of conditional cash transfer programmes: a systematic review and meta-analysis.* Journal of Development Effectiveness, 2015. **7**(3): p. 290-303.
2. McGuire, J., C. Kaiser, and A.M. Bach-Mortensen, *A systematic review and meta-analysis of the impact of cash transfers on subjective well-being and mental health in low-and middle-income countries.* Nature Human Behaviour, 2022. **6**(3): p. 359-370.
3. World Health Organization, *Global report on health equity for persons with disabilities*. 2022, Geneva: World Health Organization.
4. International Labour Organization, *World Social Protection Report 2017-19: Universal social protection to achieve the Sustainable Development Goals*. 2017, ILO: Geneva.
5. United Nations, *United Nations Convention on the Rights of Persons with Disabilities*, in *Article 1*, United Nations, Editor. 2006.

For peer review only

6. Banks, L.M., H. Kuper, and S. Polack, *Poverty and disability in low-and middle-income countries: A systematic review.* PloS one, 2017. **12**(12): p. e0189996.
7. Pinilla-Roncancio, M. and S. Alkire, *How poor are people with disabilities? Evidence based on the global multidimensional poverty index.* Journal of Disability Policy Studies, 2021. **31**(4): p. 206-216.
8. United Nations, *Disability and Development Report 2018*, D.o.E.a.S. Affairs, Editor. 2019: New York.
9. Mont, D., et al., *Estimating the Extra Costs for Disability for Social Protection Programs* 2022, UNPRPD: New York.
10. Mitra, S., et al., *Extra costs of living with a disability: A review and agenda for research.* Disability and health journal, 2017. **10**(4): p. 475-484.
11. International Labour Organization, *World Social Protection Report 2020-2022: Social protection at the crossroads - in pursuit of a better future*. 2021, ILO: Geneva.
12. Banks, L.M., et al., *Disability and social protection programmes in low-and middle-income countries: a systematic review.* Oxford Development Studies, 2016: p. 1-17.
13. Kidd, S., et al., *Leaving no-one behind: Building inclusive social protection systems for persons with disabilities*. 2019, Development Pathways: Orpington, UK.
14. Banks, L.M., et al., *The Impact of the Disability Allowance on Financial Well-Being in the Maldives: Quasi-experimental Study.* The European Journal of Development Research, 2023: p. 1-17.
15. Li, Z., et al., *The association of a disability-targeted cash transfer programme with disability status and health-care access: a quasi-experimental study using a nationwide cohort of 4· 3 million Chinese adults living with severe disabilities.* The Lancet Public Health, 2023. **8**(12): p. e933-e942.
16. Wang, H., et al., *The effect of a disability-targeted cash transfer program on universal health coverage and universal access to education: a nationwide cohort study of Chinese children and adolescents with disabilities.* The Lancet Regional Health–Western Pacific, 2023. **31**.
17. de Groot, R., et al., *The impact of the Lesotho Child Grant Programme in the lives of children and adults with disabilities: Disaggregated analysis of a community randomized controlled trial.* International Social Security Review, 2021. **74**(2): p. 55-81.
18. UNICEF. *Cash Plus*. 2021 [cited 2022 September 10]; Available from: https://www.unicef-irc.org/research/cash-plus/.
19. Banks, L.M., et al., *Disability-inclusive responses to COVID-19: Lessons learnt from research on social protection in low-and middle-income countries.* World development, 2021. **137**: p. 105178.
20. Danemayer, J., et al., *Measuring assistive technology supply and demand: a scoping review.* Assistive Technology, 2021. **33**(sup1): p. S35-S49.
21. Smythe, T., et al., *Early intervention for children with developmental disabilities in low and middle-income countries–the case for action.* International health, 2021. **13**(3): p. 222-231.
22. Roelen, K., et al., *How to make 'cash plus' work: Linking cash transfers to services and sectors.* 2017.
23. Banks, L.M., et al., *Disability-inclusive social protection research in Nepal: A national overview with a case study from Tanahun district*. 2018, London School of Hygiene & Tropical Medicine: London.

For peer review only

24. Banks, L.M., et al., *Disability-inclusive social protection research in Vietnam: A national overview with a case study from Cam Le district*. 2018, London School of Hygiene & Tropical Medicine: London.
25. Lao Statistics Bureau, *Disability Monograph of Lao PDR: From the 2015 Population and Housing Census*, M.o.P.a. Investment, Editor. 2020: Vientiane.
26. Lao Ministry of Labour and Social Welfare, *National Social Protection Strategy: Vision 2030*, D.o.P.a. Cooperation, Editor. 2020: Vientiane.
27. International Labour Organization. *Lao PDR: Situation and priorities* 2023 [cited 2024 March 1]; Available from: https://www.social-protection.org/gimi/ShowCountryProfile.action?iso=LA#:~:text=Social%20protection%20situation&text=The%20majority%20of%20the%20Lao,vulnerable%20segments%20of%20the%20population.
28. de Neubourg, C., et al., *Multiple Overlapping Deprivation Analysis (MODA).* 2012.
29. United Nations Statistics Division. *SDG indicators: Metadata repository*. 2021 [cited 2021 June 23]; Available from: https://unstats.un.org/sdgs/metadata/.
30. Varni, J.W., et al., *The PedsQL™ family impact module: preliminary reliability and validity.* Health and quality of life outcomes, 2004. **2**(1): p. 1-6.
31. Duttine, A., et al., *Assessment of the feasibility of Juntos: A support programme for families of children affected by Congenital Zika Syndrome.* Wellcome Open Research, 2022. **7**.
32. Smythe, T., et al., *The feasibility of establishing parent support groups for children with congenital Zika syndrome and their families: a mixed-methods study.* Wellcome Open Research, 2021. **6**(158): p. 158.
33. Varni, J.W., *Scaling and Scoring of the Pediatric Quality of Life Inventory™ (PedsQL).* 2014, Mapi Research Trust: Lyon, France.
34. Harvey, A.S. and M.E. Taylor, *Activity settings and travel behaviour: A social contact perspective.* Transportation, 2000. **27**: p. 53-73.
35. Moore, G.F., et al., *Process evaluation of complex interventions: Medical Research Council guidance.* bmj, 2015. **350**.
36. Zou, G., *A modified poisson regression approach to prospective studies with binary data.* American journal of epidemiology, 2004. **159**(7): p. 702-706.
37. Banks, L.M., et al., *Disability and social protection programmes in low-and middle-income countries: a systematic review.* Oxford Development Studies, 2017. **45**(3): p. 223-239.
38. Abdoulayi, S., et al., *Malawi Social Cash Transfer Programme Endline Impact Evaluation Report*. 2016, Univeristy of North Carolina at Chapel Hill & University of Malawi: Chapel Hill, North Carolina.

For peer review only

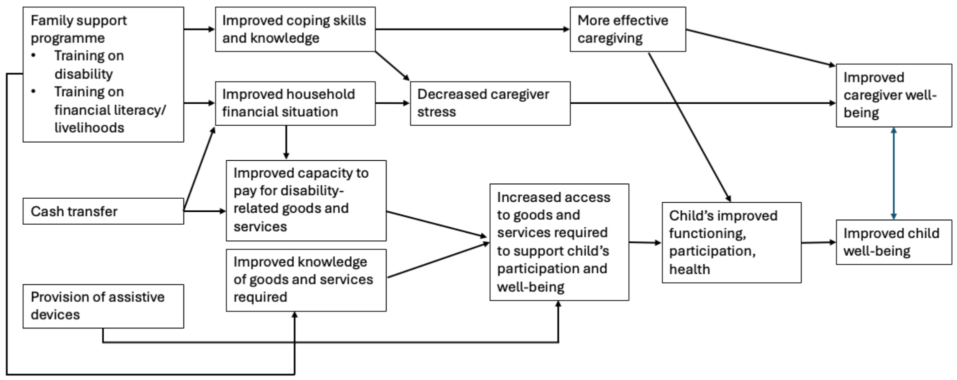

Figure 1. Theory of change for cash-plus programme

421x183mm (72 x 72 DPI)

Domain	Measure of deprivation
Education and early child development	Child (0-2): Child does not have any books/toys OR has inadequate supervision[1]<br>Child (3-5): Child is not in pre-primary/early childhood education OR has inadequate supervision<br>Child (6-17): Child is not attending school OR has missed more than 5 days of school in the last month
Health	All ages: Child has an unmet need for a health/rehabilitation service
Food security	Child (0-4): Child lives in a household that is food insecure<br>Child (5-17): Child lives in a household that is food insecure OR child reports being hungry but not eating because of lack of food in the last 30 days
Social participation	Child (0-4): Child has not been brought to a community/social event in which other children are present in the last month<br>Child (5-17): Child has at least one friend outside of their family OR did not play with friend(s) in the last 2 weeks
WASH[2]	Child (0-4): Child lives in a household without clean water source<br>Child (5-17): Child lives in a household without clean water sources OR child uses a different source than the rest of the family and this source is not a clean source OR child cannot access enough water to meet daily needs
	Child (0-4): Child lives in a household without an improved sanitation source<br>Child (5-17): Child lives in a household without an improved sanitation source sources OR child uses a different source than the rest of the family and this source is not improved OR child cannot use facility hygienically (i.e., without coming into contact with faeces, urine)
Violence	Child experiences any form of violence or discrimination in the past month

[1] Inadequate supervision defined as being left alone or under supervision of a child younger than 10 for more than one hour at least once in the last week (from UNICEF Multiple Cluster Indicator Surveys)
[2] Definitions of clean water and sanitation source according to SDG indicators (United Nations Statistic Division, 2021) and the Quality of WASH access questionnaire (Banks et al, 2019).

For peer review only

BMJ Open

Impact evaluation of a cash-plus programme for children with disabilities in the Xiengkhouang Province in Lao PDR: Study protocol for a non-randomised control trial

Journal:	*BMJ Open*
Manuscript ID	bmjopen-2023-081536.R2
Article Type:	Protocol
Date Submitted by the Author:	26-Apr-2024
Complete List of Authors:	Banks, Lena Morgon; London School of Hygiene & Tropical Medicine<br>Soukkhaphone, Bounhome; Laos Tropical & Public Health Institute<br>Scherer, Nathaniel; London School of Hygiene & Tropical Medicine<br>Siengsounthone, Latsamy; Laos Tropical & Public Health Institute<br>Carew, Mark T.; London School of Hygiene & Tropical Medicine<br>Shakespeare, Tom; London School of Hygiene and Tropical Medicine Faculty of Infectious and Tropical Diseases<br>Chen, Shanquan; London School of Hygiene & Tropical Medicine<br>Davey, Calum; London School of Hygiene and Tropical Medicine<br>Goyal, Divya; Independent consultant<br>Zinke, Anja; London School of Hygiene and Tropical Medicine, Department of Global Health & Development<br>Kuper, Hannah; The London School of Hygiene & Tropical Medicine, Clinical Research<br>Chanthakoumane, Ketmany; Laos Tropical & Public Health Institute
**Primary Subject Heading**:	Global health
Secondary Subject Heading:	Evidence based practice
Keywords:	PUBLIC HEALTH, Community child health < PAEDIATRICS, Disabled Persons, Social Support, Rehabilitation medicine < INTERNAL MEDICINE

SCHOLARONE™ Manuscripts

Impact evaluation of a cash-plus programme for children with disabilities in theXiengkhouang Province in Lao PDR: Study protocol for a non-randomised control trial

Lena Morgon Banks[1*Ω], Bounhome Soukkhaphone[2*], Nathaniel Scherer[1], Latsamy Siengsounthone[2], Mark T. Carew[1], Tom Shakespeare[1], Shanquan Chen[1], Calum Davey[1], Divya Goyal[3], Anja Zinke-Allmang[4], Hannah Kuper*[1], Ketmany Chanthakoumane[2]*

*These authors contributed equally to the work

[Ω]corresponding author: morgon.banks@lshtm.ac.uk

[1]International Centre for Evidence in Disability, London School of Hygiene & Tropical Medicine, London, United Kingdom, WC1E 7HT
[2]Laos Tropical & Public Health Institute, Vientiane, Laos
[3]Independent consultant, India
[4]Department of Population Health, London School of Hygiene & Tropical Medicine, London, United Kingdom, WC1E 7HT

For peer review only

Abstract

Introduction

More than 170 countries have implemented disability-targeted social protection programmes, although few have been rigorously evaluated. Consequently, a non-randomised control trial is being conducted of a pilot ‘cash-plus’programme implemented by UNICEF Laos and the Laos government for children with disabilities in the the Xiengkhouang Province in Laos. The intervention combines a regular cash transfer with provision of assistive devices and access for caregivers to a family support programme.

Methods and analysis

The non-randomised control trial will involve 350 children with disabilities across 3 districts identified by programme implementers as eligible for the programme (intervention arm). Implementers have also identified approximately 180 children with disabilities in neighboring districts, who would otherwise meet eligibility criteria but do not live in the project areas (control arm)The trial will assess the impact of the programme on child well-being (primary outcome), as well as household poverty, caregiver quality of life and time use(secondary outcomes). Baseline data is being collected May-October 2023, with endline 24 months later. Analysis will be intention to treat. A complementary process evaluation will explore the implementation, acceptability of the programme, challenges and enablers to its delivery and mechanisms of impact.

Ethics and dissemination

The study has received ethical approval from the London School of Hygiene & Tropical Medicine and the National Ethics Committee for Health Research in Laos. Informed consent and assent will be taken by trained data collectors. Data will be

For peer review only

collected and stored on a secure, encrypted server and its use will follow a detailed data management plan. Findings will be disseminated in academic journals and in short briefs for policy and programmatic actors, and in online and in-person events.

Strengths and limitations of this study:

- The study design is robust, combining a non-randomised control trial and a process evaluation, to assess if and how a novel cash-plus programme impacts children with disabilities and their households' wellbeing.
- Trial design involved feedback from multiple stakeholders to ensure outcomes being investigated are in line with the programme's intended aims.
- Still, individuals could not be randomised and so there may be unobservable differences between intervention and control arms.
- Similarly, the sample size likely will not have sufficient power to explore meaningful differences amongst children with disabilities (e.g., if impacts differ by the child's gender, disability type etc).

Registration: This trial is registered with the ISRCTN registry (ISRCTN80603476) https://www.isrctn.com/ISRCTN80603476. It is protocol version 5 (June 20, 2023).

Keywords: disability, cash transfer, social protection, Laos, children with disabilities

For peer review only

Introduction

Social protection is an important tool for reducing poverty and protecting people from risks that affect well-being across the lifecycle [1, 2]. People with disabilities, who comprise 16% of the global population, are a core target group for social protection [3, 4]. People with disabilities have a right to social protection under the United Nations Convention on the Rights of Persons with Disabilities (UNCRPD) which defines disability as including "… those who have long-term physical, mental, intellectual or sensory impairments which in interaction with various barriers may hinder their full and effective participation in society on an equal basis with others" [5].

Further, people with disabilities may have a higher need for social protection. For example, they are more likely to be living in poverty compared to people without disabilities[6, 7]. People with disabilities are often excluded from opportunities for decent work, due to discrimination or earlier barriers to receiving quality education and training, while caregivers often reduce time on income-generating activities for additional caretaking responsibilities [8]. They also frequently face "extra costs" for disability-related goods and services (e.g., rehabilitation, assistive devices, personal assistance) [9]. As such, people with disabilities and their households often require a higher level of income than people without disabilities to meet both basic needs and disability-related costs[10].

Many countries have recognised the need for and right to social protection amongst people with disabilities, with over 170 countries implementing disability-targeted social assistance programmes [4].The coverage and content of these programmes

For peer review only

can vary widely: for example, only 9% of people with severe disabilities receive a disability benefit in low-income countries compared to 86% of people with severe disabilities in high income countries[11]. However, there is a lack of evidence on the impact of social protection amongst people with disabilities [12, 13]. A systematic review on social protection and disability in low- and middle-income countries published in 2017 retrieved few rigorous studies on the impact of cash transfers and other programmes amongst people with disabilities, with available evidence limited to cross-sectional or qualitative studies of self-reported impacts with no impact evaluations [12]. Since then, impact evaluations of cash transfers amongst people with disabilities have been conducted for disability-targeted programmes in the Maldives and China[14-16], as well as mainstream programmes in Malawi and Lesotho[17]. Amongst people with disabilities of all ages, impacts of cash transfers were modest and mostly concentrated on improvements in food security, health status and increased health seeking behaviour [14, 17]. Further evidence is needed on the impact of different programmes from different settings, and amongst children with disabilities and their households in particular.

Additionally, there is an increasing recognition that cash transfers are an important but insufficient tool to push people out of poverty and improve their wellbeing [18]. Cash transfers alone may be particularly insufficient for people with disabilities and their households due to the poor availability, quality, accessibility and affordability of needed goods and services [19]. For example, assistive devices – such as wheelchairs, prosthetics, hearing aids, walking canes – are essential for improvingpeople with disabilities' functioning and participation; however, in many settings, people with disabilities lack access to these devices because their supply is

For peer review only

very limited, they lack information about their utility and where to access them and available devices are of poor quality[20].Additionally, children with disabilities and their households may face other barriers to improving their wellbeing, such as discrimination and negative attitudes on disability as well as lack of inclusive health, education and other services[8]. Caregivers may lack knowledge about disability and how to best support their child with a disability, which can lead to increased caregiver stress, less effective caregiving and ultimately poorer childoutcomes[21].

As such, there is an increasing focus on developing integrated social protection or “cash plus” programmes. Cash plus programmes provide recipients with both a cash transfer as well as complementary programmes, such as health insurance and linkages to key services[22]. These programmes show particular promise for children and adults with disabilities, who often face multiple barriers in improving their livelihoods and wellbeing[8]. People with disabilities report spending cash transfers primarily on health care[23, 24], so cash plus programmes with entitlements linked to health are likely to be relevant. In China, receipt of a disability-targeted cash transfer was linked to an increased likelihood of using rehabilitation and medical services amongst both children and adults with disabilities [15, 16].

Consequently, this study will conduct an impact evaluation of a cash plus programme targeted to children with disabilities in Laos. According to the 2015 Population and Housing Census, 2.8% of the population over 5 years of age in Laos is living with a disability, with prevalence higher in remote areas[25]. The Lao PDR Constitution and the Law on Persons with Disabilities stipulates equal protections of the rights of people with disabilities, andthe latter calls for the creation of a welfare fund for people with disabilities [26]. Overall, Laos’ social protection system is nascent, but growing. For example, health insurance coverage expanded rapidly

For peer review only

from 11% in 2008 to 93% currently[27]. It covers a range of preventative, promotive, curative and some rehabilitation services. However, it is acknowledged that insufficient state financing of the health system has led to inadequate services and high out-of-pocket payments[27]. Laos does not currently have comprehensive social protection programmes for people with disabilities, although expanding services and benefits to this group is listed as priority for action in the most recent National Social Protection Strategy [26].

This impact evaluation will assess a pilot of one such programme. The pilot programme combines a regular cash transfer with an assessment for and provision of rehabilitation and assistive devices. Caregivers are also invited to join a family support programme, which focuses on improving their livelihood skills and on learning more about disability and how to best support their child with a disability. The cash plus programme will be run by UNICEF Laos in partnership with the Laos government in three districts of the Xiengkhouang Province in Lao PDR. An accompanying process evaluation will also explore the implementation process, acceptability of the programme, challenges and enablers to its delivery and mechanisms of impact. This information will be critical for the Lao PDR government, given their prioritisation of disability within strategies for scaling-up the national social protection system [26]. It will also have implications for other contexts, given the lack of evaluations on disability-targeted cash-plus programmes.

Objectives

The overall aim of this research is to assess the impact of a cash-plus programme amongst children with disabilities and their households in Laos.

For peer review only

Specific objectives include:

- To evaluate the impact of a cash-plus programme on children's well-being
- To assess the impact of a cash-plus programme on caregivers' well-being
- To measure the impact of a cash-plus programme on household consumptionexpenditures
- To estimate the impact of a cash-plus programme on unmet needs for disability-related goods and services
- To explore the perceived impact,and acceptability of the cash-plus programme amongst children with disabilities and their caregivers
- To examine the implementation process, challenges and enablers to the delivery and mechanisms of impact of the cash-plus programme from the perspective of children/caregivers and implementers

Methods

Study design and settings

The trial will be a superiority, non-randomised control trial with two parallel groups. Three districts (Pek, Khoun, Phoukoud) in the Xiengkhouang Province were selected by UNICEF Laos and the Laos government to implement the cash-plus programme. Two other neighbouring districts were selected as control areas (Nonghed, Kham) after consultation between the research team (London School of Hygiene & Tropical Medicine – LSHTM, Laos Tropical & Public Health Institute – Laos TPHI) with UNICEF Laos as these areas have similar sociodemographic characteristics to the intervention districts.

A non-randomised control trial design was used as it was not possible to randomise

For peer review only

children to receiving or not receiving the cash-plus programme, as the programme implementers had logistical constraints that meant they could only operate the programme in the selected areas at this time.

Baseline data is being conducted between May to October 2023. Collection of data from the intervention group was completed before they began receiving any part of the programme. Allocation into the programme was known by implementers and the research team *a priori* although participants were not aware that they would be offered enrolment into the programme before baseline data collection. Delivery of the intervention began in September 2023, and parts (i.e. cash transfer) will be continued indefinitely funding dependent. Endline data collection will take place 24 months from baseline. The process evaluation will be conducted in 2024 after the delivery of the family support programme and assistive device components.

Intervention description

The intervention includes a cash transfer along with an assessment for assistive devices (e.g., wheelchair, prosthesis, hearing aid, walking cane). Children found to have an unmet need for assistive devices will be provided with devices free of charge as part of the programme. Caregivers will also be invited to join a family support programme, which aims to improve their understanding of their child's disability and on how to provide support to their child.Figure 1 presents a theory of change for how the intervention components are expected to improve child and caregiver well-being.

Figure 1. Theory of change for cash-plus programme

For peer review only

The programme is being delivered by UNICEF Laos and the Laos government. They currently have funding to pilot the programme for approximately 450 children with disabilities in the Pek, Phoukoud and Khoun districts of Xiengkhouang Province. The intervention is expected to continue to be offered to participants for the duration of the study period.This timeline may change if implementers face budgetary, political or other constraints in continuing to operate all or part of the programme. Funding is guaranteed until June 2024, and will be reassessed for continuation before then. Participants are free to disenroll from the programme at any time. Participants in either arm are free to take up any other programme that is offered to them during the study period.

Eligibility criteria and recruitment

Data was collected from children with disabilities and their caregivers. To be eligible for this study, children had to be living in one of the study districts at the time of baseline data collection, be 18 years of age or younger and be identified by UNICEF Laos/Laos government as having a disability. Disability determination and identification for both the intervention and control groups was conducted by UNICEF Laos. An initial sample – which strove to identify all children with disabilities in the study areas –involved a combination of past survey datashared by the government of Laos andidentification conducted by community leaders; these children were then verified by UNICEF to ensure they had a disability and were of the target age. Further details on the identification process will be explored as part of the process evaluation.

For the process evaluation, approximately 15 caregivers of children with disabilities from the intervention group will be interviewed. They will be selected to maximise

For peer review only

heterogeneity by the child's gender, disability type, age and location. Additionally, about 12programme implementers will be interviewed. They will be selected to reflect diversity of roles in the design and delivery of the programme.

Participants (primarily caregivers of children with disabilities) were invited to participate in this study by members of the research team through either in-person or phone communication. Participants were provided with, or read out, an information sheet detailing the study protocol and risks and benefits of participating. Participants were provided with compensation to offset their time and other expenses (e.g., phone data) of taking part in the research.

Outcomes

Primaryoutcomemeasures

The primary outcome measure is child wellbeing, which will be measured across multiple dimensions. Multidimensional Overlapping Deprivation Analysis (MODA) have often been used for this purpose[28]. These indicators are also in line with Sustainable Development Goal (SDG) Indicator 1.2.2, which focuses on reducing poverty "in all its forms"[29].

MODAs are indexes that collate several variables on deprivation. They can be tailored for the context and population group of interest [28]. The index for this studywas created to be relevant to children with disabilities in Laos across the years of childhood (0-17 years) and covers domains that could be feasibly affected by receipt of the cash-plus programme. (Appendix 1).It included commonly used measures, such as ones used in the Multiple Indicator Cluster Surveys that have been administered in over 100 countries including Laos.It was defined through

For peer review only

consultations with UNICEF Laos, Laos government and representatives of Organisations of Persons with Disabilities in Laos. Each indicator will be given equal weight such that the total of the index falls between 0 to 1 (0 being child experiences no deprivation on any indicator, 1 being child experiences deprivation across all indicators).

Secondaryoutcomemeasures

We will explore household consumption expenditures across food and non-food items. We will compare differences in overall consumptionexpenditures and by category (food, non-food, health, education). If possible, poverty will be measured by comparing consumption expenditures against the national poverty line. This measure is in line with SDG Indicator 1.2.1 [29].

Caregiver well-being will be measured using the Pediatric Quality of Life (PedsQL) Family Impact Module, which looks at caregiver and family strain linked to having a child with a health condition [30]. It has been used in different settings for measuring well-being of caregivers of children with disabilities [31, 32]. A score will be computed according to the tool's scoring guidance [33]. Caregiver time use across different activities will be assessed using a modified version of the 'stylised activity list' developed for the Living Standard Measurement Survey [34].

Finally, we will assess unmet need for different disability-related goods and services by creating a module on the caregiver-reported need for and use of different goods and services required to support their child's participation (e.g. healthcare, assistive devices, inclusive education supports, personal assistance). For each item,

For peer review only

caregivers will be asked if they think their child needs the good/service, if they have gone for the service and their satisfaction with that service.

Processevaluation

The process evaluation will follow the Medical Research Council (MRC) framework[35]. It will examine the structures and resources used to deliver the intervention and what was delivered, for example in terms of whether the intervention reached all whom it was designed to reach.

It will also explore mechanisms of impact, including participants' reflections on the strengths and challenges of different components and their perceptions of the impact of the programme (or lack thereof) on their lives, children and personal wellbeing. The latter will complement the quantitative measures of impact, as concepts such as well-being are complex and challenging to measure. Finally, the process evaluation will explore the role of context, for examplehow underlying socioeconomic factors, social attitudes to disability and broader disability inclusion in the study areas may have influencedthe effectiveness of the programme.

Sample size

The primary outcome measure is child well-being, measured through a MODA index. Disability-sensitive MODAs are lacking, but using data from a previous study in Vietnam as parameters [15], we assume an average score of 0.4 (score out of 1) and a standard deviation of 0.12. With a sample size of 330 in the intervention arm and 180 in control, we can detect a minimum change in score of 0.035 (1/3 of a standard deviation) with power of 80%, type 1 error of 5% and 20% loss to follow-up.

For peer review only

The process evaluation will include in-depth interviews with about 15 caregivers of children with disabilities and 12 implementers.

Data collection and management

Baseline data will be collected between May-October 2023. Endline data will be collected 24 months later.All outcomes will be collected through a mobile survey surveycreated through Open Data Kit (ODK) to promote data quality. The survey tool has built-in skips andgenerates error messages for missing data and for non-logical answers (e.g., answers to daily time use questions exceeding 24 hours). The survey was developed by researchers at LSTHM and Laos TPHI, using validated tools (e.g., PedsQL, time use adapted from Living Standards Measurements Surveys) where possible. It was translated from English into Lao and Hmong. The translated versions were reviewed by multiple native speakers and pilot tested prior to implementation.

The survey will be administered by trained data collectors, who underwent a weeklong training in the study protocols, practice using the questionnaire and on how to promote quality data collection. They are predominantly public health students at the Lao TPHI and speak Lao and/or Hmong.

The child with disabilities' primary caregiver will be the main respondent, although children with disabilities ages 5-17 may answer certain modules (e.g.,child's social participation, experiences in school). Other household members besides the primary caregiver may also contribute to sections on household consumption expenditures if they are the most knowledgeable household member.Multiple means of contacting participants were collected during baseline to decrease loss to follow-up by endline.

For peer review only

The process evaluation will be conducted in 2024 after the completion of the family support programme and delivery of assistive devices. In-depth interviews with caregivers and implementers will be conducted by trained interviewers in Lao, Hmong or English depending on the preference of the participant. They will usesemi-structured interview guides. For implementers, these guides will be adapted to reflect the expertise and role of the respondent.

Data management

Data for the trial will be collected and stored on a secure and encrypted ODK server, hosted by LSHTM. In-depth interviews will be recorded, translated and transcribed. All data will be cleaned and anonymised by an experienced data manager at Lao TPHI. Hard copies of data (e.g. written consent forms) will be kept in locked, secure cabinets at Laos TPHI. Electronic data (e.g., survey responses, interview recordings) will be kept on a password protected computer and on secure servers. A master file linking participants' names with ID numbers, signed consent forms, and contact/locator information will be stored in as an encrypted file on a password protected computer that is separate from the data files.Non-anonymised data will be destroyed 2 years after study completion.

Twenty-four months after the end of the project the data may be made available on LSHTM's Data Compass if it can be done so in a way that both allows for replication and does not lead to potential identification of participants. The possible identification of participants is a concern given that a relatively small number of children are receiving the cash-plus programme: combiningbasic details such as age, gender,

For peer review only

disability type and area of residence may be sufficient to identify a participant. The Data Protection Office at LSHTM will be consulted about the risk of identification.

If access to the data is possible, it will be made available upon request, provided the requester has a legitimate purpose for using the data. Requests will be managed by LSHTM's Data Compass team, which is independent to the research team.

Patient and public involvement

Preliminary outcome indicators were checked with the Lao Disabled People's Association, an Organisation of Persons with Disabilities, to confirm relevance for people with disabilities in Laos. Findings from the study will be disseminated back to participants (e.g., through community events).

Analysis

A detailed analysis plan will be published before the endline survey. The analysis will estimate intention-to-treat (ITT) effects.

We will assess the quality of the balance between control and intervention groups by describing the primary and secondary outcomes and sociodemographic variables at baseline. If there is evidence of imbalance, based on subjective interpretation of the magnitude of the difference, then we will plan *a priori* to include such variables in the main analysis of the primary and secondary outcomes.

We will estimate the effect of the intervention by comparing the proportions (e.g., proportion of children deprived on each domain of the MODA) and the

For peer review only

means/medians (e.g., total MODA score, household consumption expenditures) between the arms of the trial. We will report the unadjusted estimated effects as risk ratios for binary outcomes and difference in the means for continuous outcomes. In the final analysis, to increase the precision of the estimates and reduce the risk of bias from imbalances at baseline, we will use regression to adjust for the baseline levels of the outcome, stratification variables, and variables considered to be imbalanced at baseline. For binary outcomes we will model the risk ratio with a modified Poisson regression model [36]. For continuous outcomes we will use linear regression. We will disaggregate findings by genderand other characteristics if the final sample sizes are sufficiently powered.

For the process evaluation, thematic analysis will be used (Braun & Clarke, 2006). Transcripts of in-depth interviews will be analysedboth deductively and inductively in NVivo. Deductive analysis willusea coding frameworkbased on the core areas of the MRC process evaluation framework, with inductive coding being used to capture additional themes identified by analysts in the data. Analysis will consider differences by sub-groups, such as by the gender or disability type of the child.

Methods in analysis to handleprotocol non-adherenceandanystatisticalmethods to handlemissing data

Analysis will be intention-to-treat. The programme implementers do not have plans to expand the delivery of the cash-plus programme to the control areas or to offer the programme to people newly moving into the intervention areas during the study period, so spill-over is unlikely. In the unlikely event that the programme is expanded to control areas, we may reduce the period of follow-up.

For peer review only

Missing data is expected to be minimal given the quality checks in the questionnaire (i.e., built-in skip patterns, error messages for unentered data). Some remaining missing data can be imputed. For example, for consumption expenditures data, missing items or items where participants could not give an accurate estimate of value will be imputed based on the medianreported cost of that item from other households in the area.

Ethics and dissemination

Ethical approval has been received from the institutional review boards at the London School of Hygiene and Tropical Medicine in the United Kingdom (Reference: 28234) and from the National Ethics Committee for Health Research in Laos (Submission ID:2023.03).Any amendments will be approved by these bodies, and uploaded into the trial registry (https://www.isrctn.com/ISRCTN80603476).

Informed consent and assent will be sought before the start of all interviews (i.e. survey, qualitative in-depth interviews) by trained data collectors. Consent will be sought from all adults (aged 18+) providing data. Caregivers who are married and between 14-17 years will also provide their own consent per Laos law and Laos ethics protocols. Data collection with children (aged 0-17, or 14-17 and unmarried) will require both child assent and caregiver consent.

Ethical approval was granted for both in-person and remote data collection and consent taking. In-person data collection is the main and preferred strategy. However, remote data collection is needed in some instances as severe weather events and poor roads limited access by the research team to participants. So far, response rates for remote data collection have been adequate. For in-person data

For peer review only

collection, the information sheet will be either read aloud or given to the participant to read and written consent will be sought (signature or thumbprint if illiterate). For remote data collection (e.g., by phone/video conferencing), an information sheet will be read aloud to participants. Oral consent will then be sought, and recorded (on a separate file to any interviews). Children will be read a simplified information sheet and will provide written or oral recorded assent (for in-person and remote interviews respectively). The informed consent and assent processes will make it clear that the decision to participate, to withdraw or to refuse to answer any questions will not have any negative consequences, including on any pre-existing or future services they are receiving from UNICEF, the Laos government or any other provider. Further, participants' responses and their decision to participate or not will not be shared with UNICEF, the government or anyone outside of the research team.

Collected data will be anonymised using a unique participant ID. A link log which links the unique ID to name will be kept on a secure server in a password protected file, separate from the data. All study staff working with the data have undergone ethics training and will sign a confidentiality agreement. The study will comply with the General Data Protection Regulation, which requires that personal data must not be kept as identifiable data for longer than necessary for the purposes concerned. We will destroy all personally identifiable data two years after the end of the study.

Preliminary findings will be shared with UNICEF Laos, the Laos government and study participants for feedback. Findings from the trial will be published in academic journals and in short briefs targeted to a non-academic audience. Online and in-person dissemination events (e.g., conferences, webinars) will be conducted in Laos, the United Kingdom and elsewhere.

For peer review only

Discussion

The UNICEF Laos/Laos government cash-plus programme for children with disabilities is an important programme to evaluate. Importantly, little information exists on the impact of social protection, particularly cash-plus programmes, amongst people with disabilities [37]. Information is particularly lacking about such programmes amongst children, as studies are often underpowered to disaggregate by age[17]. However, access of children with disabilities and their families to social protection is particularly critical, as it may promote greater access to health, education and other support services that can have knock-on effects throughout their life-course. Information about the impact of this new, pilot programme, combined with a process evaluation detailing areas for strengthening, can help to inform improved delivery of this and other programmes in the future.

Still, there are some limitations of this evaluation. First, it was not feasible to conduct a non-randomised control trial. We will check for balance between the intervention and control arms and adjust analyses if there are differences between the two groups. However, it is possible that there are unobservable differences between arms that have not been accounted for. Second, some impacts of the programme may not be apparent after a 2-year follow-up period, particularly if there are delays in the roll-out of some components to the intervention. However, other evaluations of social protection programmes have found impacts on similar outcomes during this time frame [17, 38]. Finally, some analyses, such as disaggregation by gender, may be underpowered depending on the final sample size.

For peer review only

Despite these possible limitations, this non-randomised control trial of a cash-plus programme will contribute to a limited evidence-base. Governments, international organisations and other actors have cited limited resources and evidence, as a barriers to investment in disability-targeted programmes. The lack of evidence on the effectiveness of social protection amongst people – particularly children – with disabilities hinders informed policymaking and planning, a gap which can begin to be filled this evaluation of a promising cash-plus programme.

Declarations

Acknowledgements

We are thankful to staff at UNICEF Laos, particularly Maryam Abdu andAmphayvanChanmany for their contributions in informing the study design and identifying participants.

Author contributions

LMB, BS, NS, LS, MTC, TS, SC, CD, DG, AZA, HK, KCcontributed to the design of this protocol. LMB wrote the original draft of this paper and BS, NS, LS, MTC, TS, SC, CD, DG, AZA, HK, KCcritically reviewed. LMB, BS, KC,NS, MTC,LS contributed to the development and review of study tools. HK obtained funding.

Funding

This research was funded by the United Kingdom's Foreign, Commonwealth and Development Office (PENDA project, grant PO8073). HK's salary is funded by an NIHR Global Research Professorship. LMB's time is partially covered by an Arts & Humanities networking grant (AH/X009580/1) and the Open Access fee is covered by this grant.

For peer review only

Availability of data and materials

We are investigating if it will be possible toanonymisethe data in a way that would allow for reproduction of findings without identifying participants.

Ethics approval and consent to participate

Ethical approval has been received from the institutional review boards at the London School of Hygiene and Tropical Medicine in the United Kingdom (Reference: 28234) and from the National Ethics Committee for Health Research in Laos (Submission ID:2023.03).Written or oral recorded informed consent will be obtained for all participants.

Competing interests

LMB has previously worked as a consultant for UNICEF and holds grants in which UNICEF is a collaborator. None of this workis linked to this evaluation and is through UNICEF headquarters rather than UNICEF Laos.

References

1. Kabeer, N. and H. Waddington, *Economic impacts of conditional cash transfer programmes: a systematic review and meta-analysis.* Journal of Development Effectiveness, 2015. **7**(3): p. 290-303.
2. McGuire, J., C. Kaiser, and A.M. Bach-Mortensen, *A systematic review and meta-analysis of the impact of cash transfers on subjective well-being and mental health in low-and middle-income countries.* Nature Human Behaviour, 2022. **6**(3): p. 359-370.
3. World Health Organization, *Global report on health equity for persons with disabilities*. 2022, Geneva: World Health Organization.
4. International Labour Organization, *World Social Protection Report 2017-19: Universal social protection to achieve the Sustainable Development Goals*. 2017, ILO: Geneva.
5. United Nations, *United Nations Convention on the Rights of Persons with Disabilities*, in *Article 1*, United Nations, Editor. 2006.

6. Banks, L.M., H. Kuper, and S. Polack, *Poverty and disability in low-and middle-income countries: A systematic review.* PloS one, 2017. **12**(12): p. e0189996.
7. Pinilla-Roncancio, M. and S. Alkire, *How poor are people with disabilities? Evidence based on the global multidimensional poverty index.* Journal of Disability Policy Studies, 2021. **31**(4): p. 206-216.
8. United Nations, *Disability and Development Report 2018*, D.o.E.a.S. Affairs, Editor. 2019: New York.
9. Mont, D., et al., *Estimating the Extra Costs for Disability for Social Protection Programs* 2022, UNPRPD: New York.
10. Mitra, S., et al., *Extra costs of living with a disability: A review and agenda for research.* Disability and health journal, 2017. **10**(4): p. 475-484.
11. International Labour Organization, *World Social Protection Report 2020-2022: Social protection at the crossroads - in pursuit of a better future*. 2021, ILO: Geneva.
12. Banks, L.M., et al., *Disability and social protection programmes in low-and middle-income countries: a systematic review.* Oxford Development Studies, 2016: p. 1-17.
13. Kidd, S., et al., *Leaving no-one behind: Building inclusive social protection systems for persons with disabilities*. 2019, Development Pathways: Orpington, UK.
14. Banks, L.M., et al., *The Impact of the Disability Allowance on Financial Well-Being in the Maldives: Quasi-experimental Study.* The European Journal of Development Research, 2023: p. 1-17.
15. Li, Z., et al., *The association of a disability-targeted cash transfer programme with disability status and health-care access: a quasi-experimental study using a nationwide cohort of 4· 3 million Chinese adults living with severe disabilities.* The Lancet Public Health, 2023. **8**(12): p. e933-e942.
16. Wang, H., et al., *The effect of a disability-targeted cash transfer program on universal health coverage and universal access to education: a nationwide cohort study of Chinese children and adolescents with disabilities.* The Lancet Regional Health–Western Pacific, 2023. **31**.
17. de Groot, R., et al., *The impact of the Lesotho Child Grant Programme in the lives of children and adults with disabilities: Disaggregated analysis of a community randomized controlled trial.* International Social Security Review, 2021. **74**(2): p. 55-81.
18. UNICEF. *Cash Plus*. 2021 [cited 2022 September 10]; Available from: https://www.unicef-irc.org/research/cash-plus/.
19. Banks, L.M., et al., *Disability-inclusive responses to COVID-19: Lessons learnt from research on social protection in low-and middle-income countries.* World development, 2021. **137**: p. 105178.
20. Danemayer, J., et al., *Measuring assistive technology supply and demand: a scoping review.* Assistive Technology, 2021. **33**(sup1): p. S35-S49.
21. Smythe, T., et al., *Early intervention for children with developmental disabilities in low and middle-income countries–the case for action.* International health, 2021. **13**(3): p. 222-231.
22. Roelen, K., et al., *How to make 'cash plus' work: Linking cash transfers to services and sectors.* 2017.
23. Banks, L.M., et al., *Disability-inclusive social protection research in Nepal: A national overview with a case study from Tanahun district*. 2018, London School of Hygiene & Tropical Medicine: London.

For peer review only

24. Banks, L.M., et al., *Disability-inclusive social protection research in Vietnam: A national overview with a case study from Cam Le district*. 2018, London School of Hygiene & Tropical Medicine: London.
25. Lao Statistics Bureau, *Disability Monograph of Lao PDR: From the 2015 Population and Housing Census*, M.o.P.a. Investment, Editor. 2020: Vientiane.
26. Lao Ministry of Labour and Social Welfare, *National Social Protection Strategy: Vision 2030*, D.o.P.a. Cooperation, Editor. 2020: Vientiane.
27. International Labour Organization. *Lao PDR: Situation and priorities* 2023 [cited 2024 March 1]; Available from: https://www.social-protection.org/gimi/ShowCountryProfile.action?iso=LA#:~:text=Social%20protection%20situation&text=The%20majority%20of%20the%20Lao,vulnerable%20segments%20of%20the%20population.
28. de Neubourg, C., et al., *Multiple Overlapping Deprivation Analysis (MODA).* 2012.
29. United Nations Statistics Division. *SDG indicators: Metadata repository*. 2021 [cited 2021 June 23]; Available from: https://unstats.un.org/sdgs/metadata/.
30. Varni, J.W., et al., *The PedsQL™ family impact module: preliminary reliability and validity.* Health and quality of life outcomes, 2004. **2**(1): p. 1-6.
31. Duttine, A., et al., *Assessment of the feasibility of Juntos: A support programme for families of children affected by Congenital Zika Syndrome.* Wellcome Open Research, 2022. **7**.
32. Smythe, T., et al., *The feasibility of establishing parent support groups for children with congenital Zika syndrome and their families: a mixed-methods study.* Wellcome Open Research, 2021. **6**(158): p. 158.
33. Varni, J.W., *Scaling and Scoring of the Pediatric Quality of Life Inventory™ (PedsQL).* 2014, Mapi Research Trust: Lyon, France.
34. Harvey, A.S. and M.E. Taylor, *Activity settings and travel behaviour: A social contact perspective.* Transportation, 2000. **27**: p. 53-73.
35. Moore, G.F., et al., *Process evaluation of complex interventions: Medical Research Council guidance.* bmj, 2015. **350**.
36. Zou, G., *A modified poisson regression approach to prospective studies with binary data.* American journal of epidemiology, 2004. **159**(7): p. 702-706.
37. Banks, L.M., et al., *Disability and social protection programmes in low-and middle-income countries: a systematic review.* Oxford Development Studies, 2017. **45**(3): p. 223-239.
38. Abdoulayi, S., et al., *Malawi Social Cash Transfer Programme Endline Impact Evaluation Report*. 2016, Univeristy of North Carolina at Chapel Hill & University of Malawi: Chapel Hill, North Carolina.

For peer review only

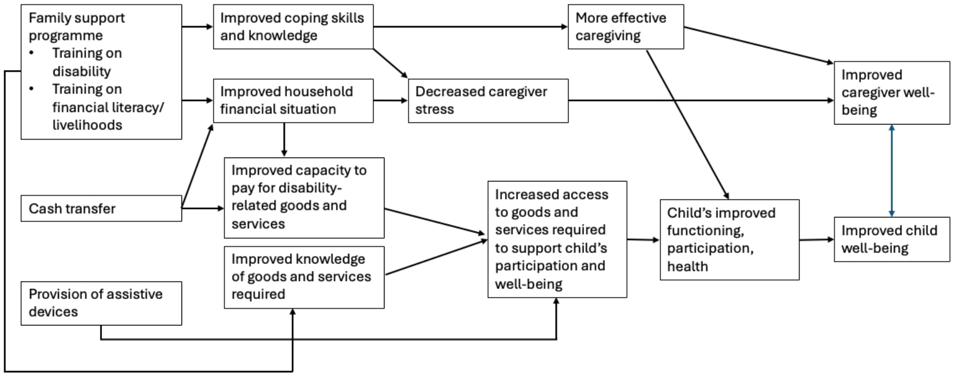

Figure 1. Theory of change for cash-plus programme

421x183mm (72 x 72 DPI)

Domain	Measure of deprivation
Education and early child development	Child (0-2): Child does not have any books/toys OR has inadequate supervision[1]<br>Child (3-5): Child is not in pre-primary/early childhood education OR has inadequate supervision<br>Child (6-17): Child is not attending school OR has missed more than 5 days of school in the last month
Health	All ages: Child has an unmet need for a health/rehabilitation service
Food security	Child (0-4): Child lives in a household that is food insecure<br>Child (5-17): Child lives in a household that is food insecure OR child reports being hungry but not eating because of lack of food in the last 30 days
Social participation	Child (0-4): Child has not been brought to a community/social event in which other children are present in the last month<br>Child (5-17): Child has at least one friend outside of their family OR did not play with friend(s) in the last 2 weeks
WASH[2]	Child (0-4): Child lives in a household without clean water source<br>Child (5-17): Child lives in a household without clean water sources OR child uses a different source than the rest of the family and this source is not a clean source OR child cannot access enough water to meet daily needs
	Child (0-4): Child lives in a household without an improved sanitation source<br>Child (5-17): Child lives in a household without an improved sanitation source sources OR child uses a different source than the rest of the family and this source is not improved OR child cannot use facility hygienically (i.e., without coming into contact with faeces, urine)
Violence	Child experiences any form of violence or discrimination in the past month

[1] Inadequate supervision defined as being left alone or under supervision of a child younger than 10 for more than one hour at least once in the last week (from UNICEF Multiple Cluster Indicator Surveys)
[2] Definitions of clean water and sanitation source according to SDG indicators (United Nations Statistic Division, 2021) and the Quality of WASH access questionnaire (Banks et al, 2019).

For peer review only
